# Modelling Gas Transport in Multiphasic Materials: Application to Semicrystalline Membranes

**DOI:** 10.3390/membranes15030076

**Published:** 2025-03-02

**Authors:** Lorenzo Merlonghi, Marco Giacinti Baschetti, Maria Grazia De Angelis

**Affiliations:** 1 Department of Civil, Chemical, Environmental and Material Engineering (DICAM), Alma Mater Studiorum–Università di Bologna, Via Terracini 28, 40131 Bologna, Italy; 2 DPI, P.O. Box 902, 5600 AX Eindhoven, The Netherlands; 3 Institute for Materials and Processes, School of Engineering, University of Edinburgh, Sanderson Building, Robert Stevenson Road, Scotland EH9 3FB, UK

**Keywords:** gas transport modelling, semicrystalline membranes

## Abstract

The description of gas permeation across heterogeneous materials has been studied with many methods, mainly focusing on composites with high aspect ratios and low filler volume fractions. In the present work, the extension of these approaches to semicrystalline polymers is studied, considering a wide range of crystalline volume fractions to tackle applications ranging from membranes to barrier materials. A numerical approach focused on tortuosity effects related to the presence of impermeable crystalline domains was considered. Algorithms based on random sequential adsorption and Voronoi tessellation were used to reproduce the morphology of semicrystalline polymers. The flux reduction across the microstructures generated due to the presence of impermeable crystals was calculated by solving local mass balance through a finite volume method. Using this strategy, it was possible to investigate the effect of crystallites’ arrangement, size distribution, orientation and shape on the relative permeability and the tortuosity of semicrystalline membranes. The results were analyzed considering existing macroscopic models and new analytical equations were proposed in order to account on such morphological effects for the prediction of the tortuosity in semicrystalline polymers.

## 1. Introduction

The permeability of gases in polymeric materials is of great interest in many research areas, spanning from barrier applications, such as packaging [1] and high-pressure gas storage [2], up to membrane separation processes [3].

For barrier applications, the main goal is to prevent gas flux from the inner core to the environment and vice versa. While in packaging, the shelf life of perishable products must be ensured by maintaining a proper atmosphere inside the package, in gas storage and transport, gas flow must be completely avoided to prevent massive leakages. This is particularly important for flammable gases such as hydrogen, regarding which a lot of research is currently ongoing in view of its possible use as an energy carrier [4]. For the latter application, in particular, semicrystalline polymers are nowadays gaining a lot of attention as liner materials for gas pipelines and storage tanks. Their low weight, high processability and ease of welding bring lower costs of transport and installation with respect to steels [5,6]. Moreover, while steels undergo embrittlement in a hydrogen atmosphere [7], some polymeric materials are not subjected to any degradation in the presence of hydrogen [8], which makes their use as liners potentially the best solution, as well as in terms of durability and recyclability. Some examples are semicrystalline polymers (SCPs) such as polyamide 6 (PA6) and high-density polyethylene (HDPE), nowadays used as liner materials for commercially available type-IV hydrogen storage tanks [2,5,9,10], while for low-pressure applications such as packaging, polymer matrix composites (PMCs) based on polyethylene and polyamide matrices filled with inorganic clay or graphite have been studied by many authors, who have demonstrated improvements in the barrier properties thanks to the addition of the inorganic impermeable phase [11,12,13,14].

On the other hand, in membrane separation processes, the gaseous flux of each component across the material needs to be tailored in order to meet process specifications and increase separation efficiency, primarily driven by selective permeability. The process is not only environmentally friendly, often requiring less energy than traditional methods, but also highly adaptable and versatile, considering different membrane module architectures. Semicrystalline membranes have garnered significant attention in gas separation applications due to their unique structural properties, which combine both crystalline and amorphous phases. This dual-phase architecture enables semicrystalline membranes to offer an appealing balance of mechanical stability, selective permeability, and controlled diffusion pathways. While crystalline domains provide structural integrity and thermal stability, ensuring the membrane remains durable under various operational conditions, amorphous domains allow for a degree of molecular mobility, creating permeable pathways for gases. By manipulating their crystallinity and morphology, in principle, it is possible to tailor these membranes for specific gas separation tasks. Some examples are PA6 [15], polyethylene oxide (PEO) [16], Pebax [17,18], styrene-ethylene oxide (SEO) block copolymers [19], and semicrystalline mixed matrix membranes (MMMs) [3,20,21], which have gained a lot of attention in the design of membranes for gas separations processes, such as carbon dioxide capture, hydrogen purification, or methane recovery.

Generally, mass flux across dense polymeric materials is quantified by permeability, which is related to diffusivity and solubility [22]. Such parameters depend on the physicochemical features of the polymer and of the gas, as well as on the interaction between them [23]. In particular, the density, free volume, crystallinity, thermal history and morphology of the polymer as well as its chemical nature have to be known and controlled in order to optimize the barrier properties or the separation efficiency with respect to a given gaseous mixture. In particular, in multiphasic systems such as SCPs, PMCs and semicrystalline MMMs, polymeric crystallites and/or inorganic fillers are usually organized in a dispersed phase, embedded in a continuous and amorphous polymeric matrix.

As a consequence of the fact that both crystallites and fillers are impermeable to gases with respect to the continuous phase, the mass transport in these materials is often hindered with respect to the completely amorphous or unloaded polymer. In particular, these materials usually show a reduction in the solubility, which is proportional to the volume fraction of the discontinuous phase, and in the diffusivity, which is lowered by the increased tortuosity of the diffusive path travelled by the gaseous molecules, forced to pass around the impermeable or semipermeable domains embedded in the matrix [24]. As a result, diffusivity is not only affected by the volume fraction of such domains, but also by their aspect ratio, shape and orientation [25,26,27,28,29,30]. The microstructure and the distribution of the discontinuous phase is therefore extremely important in defining the transport properties of these heterogeneous materials.

In the case of PMCs, impermeable inorganic particles with a high aspect ratio, up to 100, like nanocomposites loaded with graphene [11] or clays [13], are usually considered for barrier applications, thanks to their ability to lower permeability even at low filler volume fractions, while semi-permeable and selective fillers such as zeolites [31] or metal organic frameworks, MOFs [32], are considered in MMMs for membrane separation processes [3]. However, producing them is challenging: the frequent aggregation of fillers can diminish the material performance by reducing the barrier performance of PMCs or the selectivity of MMMs. Moreover, poor compatibility between the polymer and the fillers in PMCs may lead to defects at the interface, which strongly decrease the efficiency of the materials, as they represent a shortcut for the diffusing molecules [12]. In some particular PMCs, it was also found that the presence of the fillers leads to an increase in the free volume of the amorphous phase near the interface, so that the tortuous effect is more than counterbalanced, leading to an increase in the permeability [33]. Other authors observed a similar effect in the solubility, but not on the diffusivity, so the overall permeability still decreased due to the presence of the fillers [14,34].

In the case of SCPs, crystalline and amorphous chains are covalently bonded, having the same chemical nature. In this case, therefore, compatibility is ensured, and voids and defects are generally avoided. For these reasons, SCPs such as PA6 are preferred nowadays to PMCs in designing high-pressure resistant liner materials for gas storage applications [5,6], but have also been studied as membranes for gas separation processes at high pressures, combining high mechanical strength and good separation performance [15]. However, the real microstructure of bulk crystallized SCPs is very complex, extending on different length scales: lamellar crystallites of nanometric thickness can grow radially from the same nucleation points to form spherulitic domains, with diameters in the order of micrometers. Moreover, in highly crystalline materials, spherulites can appear as dense grains [35,36]. Typically, while the lamellar thickness is in the order of nanometers, their width is in the order of a few microns, so they are expected to show a very high aspect ratio, in the order of 10–100 [37,38,39,40,41]. On the other hand, spherulites’ dimensions can range from 1 up to 100 microns, but their aspect ratio is limited, so they can be assumed to be spheroidal, with an aspect ratio between 0.25 and 4 [42,43,44]. In addition, different amorphous phases may exist depending on their position with respect to the crystals. Between lamellae and close to the crystal interface a semi-ordered and less mobile amorphous phase sometimes indicated as rigidified amorphous fraction (RAF) is believed to exist [45]. Other studies consider, instead, that crystals impose additional constraints on the amorphous phase thus increasing its density and decreasing its solubility [46,47].

## 2. State of the Art

In general, the properties of multiphasic materials can be derived from the properties of each phase. In particular, the problem of the description of the transport properties (energy, momentum, mass) of heterogeneous materials has been addressed in many different ways. The two main theories considered are the resistance model approach [48,49] and the effective medium theory [50,51].

The first method relies on an analogy between the current flow through a series-parallel array of resistors and the transport properties of a composite membrane made by different phases. As a result of the fact that each phase property can be associated with a resistance, it is possible to describe quite complex systems simply by coupling parallel and series resistances [48]. However, this approach entails a fictious representation of the material, far from its real morphology, and for this reason, it will not be considered in this work.

On the other hand, the effective medium theory is based on the combination of the properties of different phases to obtain the permeability of an equivalent effective medium, by considering the overall morphology [50]. The first theory of this kind comes from a continuum analysis proposed in 1873 by Maxwell [52] who derived an analytical expression for a biphasic system containing identical spheres embedded in a continuous matrix. This approach has become very popular, and it is still used today to provide a first evaluation of the effect of the dispersed phase on the properties of a biphasic material, at low volume fractions, below 20%. Other authors also extended this method to different microstructures, like those formed by prolate and oblate ellipsoids (Wagner [53], Fricke [54,55] and Sillars [56]), or to a higher loading of particles, up to 35% (Bruggeman [57]).

Another approach, focused on impermeable, ordered and lamellar inclusions, was originally proposed by Barrer and Petropoulos in 1961 [58] and later extended by Nielsen in 1967 [59]. Other extensions such as the one proposed by Bhardwaj [29] accounted for the filler orientation with respect to the flow direction. Similar systems were also considered by Aris [60] who developed a rigorous solution for the hindered diffusion in ordered 2D systems formed by regular arrays of rectangles. This model was then extended by Cussler and coworkers to polydisperse arrays as well as random and 3D systems focusing mainly on high aspect ratio systems such as PMCs [61,62]. Later, other authors compared approaches to represent the mass transfer in binary 3D composites, considering spherical, cylindrical, and cubic arrays dispersed in a continuous matrix [50,51,63].

Other strategies, such as the ones based on the percolation theory, introduced for the first time by Broadbent and Hammersley in 1957 [64], account for the agglomeration of the fillers, so that when a certain volume fraction is reached, the dispersed phase becomes locally continuous. Typically, in such systems, the dispersed phase is the permeable/conductive one, so that when a certain percolation threshold is reached, the overall composite material becomes conductive/permeable. When the dispersed phase is impermeable, the situation is reversed, and the material becomes completely impermeable above the percolation threshold. However, as in SCPs and PMCs, the permeable phase is always above the percolation threshold; this approach is of limited interest and has not been considered further in this analysis.

Other models as well as other extensions of previous models can be found in the available literature for the description of the permeability in composite systems. It is, however, out of the scope of the present work to give a complete overview of all of them, since several bibliographic reviews exist in this concern [65,66]. In a similar way, more detailed reviews concerning analytical models for semicrystalline polymers were published by Michaels and Bixler [24] and Hedenqvist and Gedde [67], which pointed out that such systems can be described by the Fricke theory [54,55] by considering crystallites as oblate impermeable ellipsoids perpendicularly aligned with respect to the flux. However, as the Fricke theory is based on the diluted regime, in principle, it should not be applicable to crystalline volume fractions higher than 20%.

Apart from analytical models, simulation-based approaches have been recently considered in order to describe the permeation properties of systems characterized by more complex microstructures. These methods can be divided between those based on the Monte Carlo algorithm, which simulates the tortuous path walked by the molecules through a random walk analysis [68,69], and those based on the numerical solution of the transport partial differential equations in multiphasic systems through finite volumes or finite elements discretization [27,70,71]. Independently of the method chosen, the reproduction of the microstructure usually relies on random sequential adsorption (RSA) algorithms [72,73]. This method inserts one particle at a time inside the computational domain in order to reach a desired volume fraction by avoiding particle overlapping. The strategy is very simple and flexible, but it is limited by a certain maximum packing fraction, which depends on geometry, aspect ratio, orientation, and size distribution of particles [74]. This approach typically finds application in problems related to heat conduction [75,76], stress–strain response [77,78] and mass transport [25,79] but has been seldom applied to gas permeability in semicrystalline materials. In particular, these studies are mainly related to the analysis of composite materials, focusing on the analysis of 2D and 3D random distributions of fillers with different shapes and dimensions, with filler content seldom exceeding diluted conditions [27,28,65]. RSA is therefore very effective for the analysis of PMCs due to the low volume fraction of the impermeable phase, which typically never exceeds 20% [11,73,79], but it cannot be used to reach the higher volume fraction typically found in highly crystalline SCPs. In addition to that, most analytical models and simulation-based approaches, designed to describe high aspect ratio fillers in PMCs, led to an ineffective prediction of the transport properties at higher loading, where the impermeable phase volume fraction is outside the diluted regime. Therefore, the results of those studies cannot be easily transferred to the case of SCPs, characterized by lower aspect ratios and higher loadings of impermeable phase.

To overcome such limitation, another strategy will be also considered in this work, based on a tessellation of the geometry, defined as a partition of the tridimensional domain into space-filling polyhedra. The most popular algorithm that can be used to perform a tessellation is the Voronoi algorithm. By using this approach, tessellated microstructures were reproduced by different authors in order to describe the mechanical properties of grain-based polycrystalline materials [80,81]. However, to the authors’ best knowledge, no works based on numerical simulations nor analytical expressions of the relative permeability are found in the literature for such grain-based materials, and thus are included in this work.

Another approach based on numerical simulations was used by Nillson et al. [30] and Mattozzi et al. [82], which reproduced the intra-spherulitic microstructure of polyolefins, including lamellar section variations, twisting, and branching. A unique RSA algorithm was used in this case to build radial growing lamellae by adding polyhedral bricks starting from the same nucleation point. Then, the intra-spherulitic tortuosity was computed through an RWM based on a Monte Carlo algorithm. This approach was later extended to higher values of lamellae volume fractions and aspect ratios by Pokorny et al. [83,84]. However, as the microstructure reproduced using this unique approach focuses on a single spherulite rather than on the whole semi-crystalline structure, is therefore not easily applicable to the determination of large-scale tortuosity of the materials, which should include the gas diffusion between spherulites.

In this work, we aim to fill this knowledge gap by proposing a simulation-based approach based on the finite volumes method which allows to elucidate how volume fraction, aspect ratio, dispersion, size distribution and orientation of crystalline domains affect the transport properties of SCPs. In particular, in this study, only the effect of the ideal morphology was investigated, focusing on the case of impermeable inclusions, leaving to future studies the effects of the inclusions on the thermodynamic and transport properties of the amorphous matrix. In addition, the possible difference related to interfacial defects or RAF properties was neglected so that ideal SCPs could be considered similar to ideal PMCs and described with similar approaches. The analysis was performed in a low aspect ratio range, starting from moderate crystallinity systems where crystallites are assumed to be spherical, moving to highly crystalline materials in which spherulites deform in polyhedral grains, to mimic a bulk crystallized material. This would also simplify possible future extension to MMMs, characterized by semipermeable fillers, which have indeed lower aspect ratio with respect to nanocomposites for barrier applications.

The obtained results were compared with the outcomes of existing analytical models mainly derived from effective medium approaches introduced above to study their applicability to these systems and possibly inspire proper modification to improve their description of the tortuosity of SCPs.

## 3. Mathematical Background

Considering a generic polymeric membrane through which a generic gas is allowed to diffuse, the volumetric gas flux per unit area at the steady state J is constant and can be expressed as the product of the permeability of the gas in the medium P, and the gradient of the gas fugacity across the membrane ∇f, leading to the following:

(1) J=P∇f

For a completely amorphous matrix, it is therefore possible to compute the mass flux per unit area $J_{a}$ by knowing the value of ∇f and the amorphous matrix permeability $P_{a}$:

(2) $$J_{a}=P_{a}\nabla f$$

As a result, is possible to define the relative permeability $P_{r}$ as a measure of the efficiency of the biphasic material in terms of flux reduction with respect to its pure amorphous permeable phase:

(3) $$P_{r}=\frac{J}{J_{a}}=\frac{P}{P_{a}}$$

For dense polymeric membranes, the solution diffusion theory [22,85,86] is usually supposed to hold so that the permeability can be directly related to the diffusion coefficient D and to the solubility coefficient S:

(4) P=D×S

As a consequence, in the same way as performed in Equation (3), is possible to define the relative diffusivity coefficient $D_{r}$ and the relative solubility coefficient $S_{r}$ as:

(5) $$D_{r}=\frac{D}{D_{a}}$$

(6) $$S_{r}=\frac{S}{S_{a}}$$

where the coefficients ${\;D}_{a}$ and $S_{a}$ are the ones that the continuous and amorphous phase exhibit in the multiphasic material, considering possible constriction caused by the presence of the inclusions in SCPs. As said above, these properties can differ for the pure unconstrained amorphous material due to the presence of the crystals, which may modify the mobility of the free polymeric chains [24] as well as its density [46]. In the present work, those contributions are included in the term $P_{a}$, which is considered an arbitrary numerical constant to be used to scale P (obtained by solving local mass balance on the domain of interest through FVM) in order to compute $P_{r}$ trough Equation (3).

By assuming that the gas cannot be dissolved in the dispersed phase [24,87], $S_{r}$ can simply be expressed as a function of the impermeable phase volume fraction ϕ:

(7) $$S_{r}=1-\phi$$

On the other hand, as $D_{r}$ is related to the tortuous path walked by the molecules, it depends not only on ϕ, but also on its morphology, orientation, and aspect ratio. This complex dependence is usually embedded in a parameter called tortuosity factor τ [24], which is here defined as:

(8) $$\tau =\frac{1}{D_{r}}$$

Finally, by considering the solution diffusion model, $P_{r}$ can be expressed as:

(9) $$P_{r}=\frac{1-\phi }{\tau }$$

In this work, $P_{r}$ is calculated through Equation (3) by knowing J and $J_{a}$, which are computed through FVM. Then, τ is calculated through Equation (10) by knowing ϕ and $P_{r}$. Historically, different analytical expressions for τ have been proposed, depending on the geometries of interest. Some of them will be reported below; in this regard, it is important to stress that among all the models available in the literature, we selected the ones more consistent with the results obtained in the present work through FVM.

The first effective medium theory was introduced by Maxwell in 1873 [52], focusing on a model developed for dielectric polarization, considering a biphasic effective medium made by spheres of identical radii dispersed into a matrix with a different dielectric permittivity, subjected to a uniform electric field. In a diluted regime, when the volume fraction of the spheres ϕ is lower than 20%, the spheres are assumed to not interact with each other so that a simple analytical expression for the relative permittivity could be derived, which share the same mathematical formulation with the relative gas permeability $P_{r}$ of a similar material, subjected to a uniform gas fugacity gradient. For the simplest case of impermeable spheres, this analysis leads to the following expression of τ as a linear function of ϕ:

(10) $$\tau =1+\frac{1}{2}\phi$$

**Figure 1:**
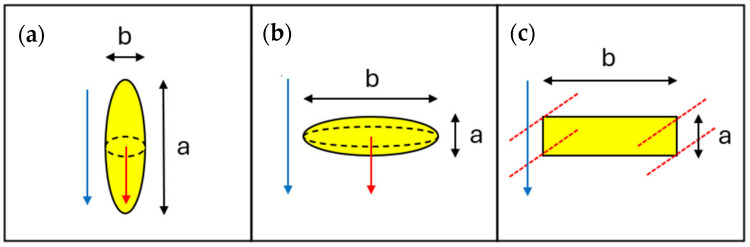
Comparison between the characteristic dimensions used to compute the aspect ratio α = a/b for different geometries: (**a**) 3D prolates, (**b**) 3D oblates and (**c**) 2D lamellae. The direction of the flux is depicted in blue. For the case of ellipsoids, the symmetry versor is depicted in red and is always parallel to the direction of the flux, while the intersection between the symmetry plane and the ellipsoid is depicted as a black dashed line. For the case of lamellae, the 3D geometry is obtained by extending the yellow area on the red dotted lines thus considering parallelepipeds of infinite width.

Other authors like Wagner in 1914 [53] extended the Maxwell analysis in order to derive an expression for the dielectric polarization that accounts for the deviation from spheres. In 1931, Fricke [54,55,63,88,89], and later in 1937, Sillars [56,90] extended the Wagner theory to suspensions of spheroidal particles. The latter models start from different visions and use different parameters, namely n and m, to describe the aspect ratio, α, of the ellipsoids, which is here defined as the ratio between one of the equal axis divided by the symmetry axis, so that α<1 for prolates and α>1 for oblates, as depicted in Figure 1a,b. Despite the different mathematical formulations, it is possible to show that the model from Fricke and Sillars (or MWS, as will be indicated in the following from Maxwell Wagner and Sillars contributions) arrives at the equivalent conclusion when considering the dependence of tortuosity from the filler loading, which remains substantially linear, as already seen in the Maxwell expression. In particular, all the different models can be described by the simplified relationship shown in Equation (11):

(11) $$\tau =1+\delta_{\alpha }\phi$$

where $\delta_{\alpha }$ is a parameter dependent on the aspect ratio of the dispersed phase α, which is related to different parameters proposed by Fricke and Sillars, respectively named m  and n, as shown in Table 1. It can be noticed that, despite the different formulations, the two models proposed by Fricke and Sillars are mathematically equivalent, as it is better discussed in Supplementary Section S.1. For this reason, only the MWS model will be considered in the following. It can be noted that for n=1/3, the MWS model is equal to the Maxwell one; for n=0, it becomes equal to the parallel resistance model (τ = 1, $P_{r}$ = 1−ϕ); and for n=1, the expression becomes equal to the series resistance model (τ tends to infinity, $P_{r}$ = 0).

Another model that includes the dependency of τ on α comes from a continuum analysis made by Nielsen [59] focusing on systems with impermeable lamellar inclusions of infinite width. According to the Nielsen model, the tortuosity is defined as the distance traveled by the molecule during diffusion in the multiphasic membrane, divided by its thickness, resulting in the following expression:

(12) $$\tau =1+\frac{\alpha }{2}\phi$$

where α is the aspect ratio of the lamellae in a 2D array, defined as the length of the lamellae a divided by the thickness b, as depicted in Figure 1c. It can be noted that for α=1 the Nielsen model is equal to the Maxwell one; for α=0, it becomes equal to the parallel resistance model; and for an α that tends to infinity, the expression becomes equal to the series resistance model, similarly to what is observed for the MWS model.

By considering the equations in Table 1, it is evident that 2D and 3D models like Nielsen and MWS can be used to describe systems which involve any dispersed impermeable phase, by using an equivalent aspect ratio, which depends on the geometry considered and thus the model used. As an example, by using the experimental tortuosity of various gases in different SCPs, it is possible to calculate the equivalent aspect ratio of impermeable crystallites, as shown in Table S12. More detailed information is given in Supplementary Section S.3 considering the determination of the tortuosity values reported in the table. Focusing on the results obtained, the equivalent aspect ratio increases with crystallinity. In particular, to obtain the same value of tortuosity, the MWS model (more reliable for real 3D systems) requires a lower aspect ratio than the Nielsen model (which assumes a less realistic 2D material).

It is clear from this analysis that for low values of *ϕ*, the equivalent aspect ratio computed using the MWS model agrees with the one that can be observed in spherulites (0.25–4) [42,43,44] but is very far from the one expected for lamellae (10–100) [37,38,39,40,41]. On the other hand, by increasing the crystallinity, an aspect ratio in the order of 10 is still required to represent the experimental tortuosity, a value which is definitely higher than those observed for spherulites.

In general, the equivalent aspect ratio falls between the one expected for spherulites and lamellae. The obtained results may also be related to the fact that the effective aspect ratio of the lamellar system is lower with respect to the one observed in single lamellae, due to the fact that lamellae are randomly oriented in real SCPs, and often organized in stacks. From another point of view, however, differences can also be related to the fact that the models considered were developed for dilute system and may give inconsistent results when used for high loadings. The linear increase in τ as a function of ϕ is indeed generally accepted for system in the limit of dilute conditions, for which ϕ≤ 20%, but when ϕ> 20%, different approaches must be used. As an example, Bruggeman [57], in an attempt to extend the Maxwell approach to systems made of spheres with volume fractions up to ϕ= 35%, obtained the following equation for tortuosity:

(13) $$\tau ={\left(1-\phi \right)}^{0.5}\;$$

From another point of view, while analyzing 2D ordered arrays made by lamellae of infinite width, other authors noticed that τ could be described by a first-order dependence on ϕ, related to the resistance in the slits between lamellae, and a second-order term accounting for the increased tortuous path around each lamella [60,62,73]. According to this theory, the following expression is obtained:

(14) $$\tau =1+\frac{\alpha \phi }{2\sigma }+\frac{{\alpha^{2}\phi }^{2}}{4(1-\phi )}$$

This model, here called the Aris model, differs from the other previously mentioned ones by the presence of the slit shape σ, a constant related to the geometry and arrangement of the inclusions, here defined as the distance between two lamellae in the same plane, divided by their thickness. It is noticed that the same analysis can be extended to 3D geometries, by simply defining σ as the ratio between the normal surface of the slit, divided by the lateral surface of the lamellae [25]. It is also noticed that by neglecting the second-order term, for σ=1, the expression becomes equal to the Nielsen model (Equation (12)). As consequence, a large deviation with respect to the Nielsen model is expected not only for high values of α typically found in PMCs, but also for high values of ϕ typically found in SCPs. On the other hand, for disordered 2D arrays, a similar second-order expression was proposed by Lape et al. [61]:

(15) $$\tau =1+\frac{2}{3}\alpha \phi +\frac{1}{9}\alpha^{2}\phi^{2}$$

It is worth noticing that all mentioned approaches describe aligned lamellae. The effect of fillers orientation was studied, for instance, by Bharadwaj [29], who proposed to that aim a modification of the Nielsen model:

(16) $$\tau =1+f_{\theta }\frac{\alpha }{2}\phi$$

where $f_{\theta }$ is defined as the orientation factor, which, for ordered 2D systems, is equal to:

(17) $$f_{\theta }=\cos^{2}\theta$$

with θ representing the orientation angle, defined as the angle between the direction of the flux and the direction of the thickness of the lamellae. As result, when the lamellae are oriented parallel to the flux, the expression becomes equal to the parallel resistance model, while when the fillers are oriented perpendicular to the flux, the expression becomes equal to the original Nielsen model.

In addition, it is also possible to compare the expressions previously proposed with the one derived by Michaels and Bixler [10] for the special case of SCPs:

(18) $$\tau =1+\phi \left[\frac{0.384+{\left(0.785-\frac{1}{\alpha }\right)}^{2}}{1.848-3{\left(0.785-\frac{1}{\alpha }\right)}^{2}}\right]$$

Also in this case, the expression of τ as function of ϕ is linear, according with Equation (12), where the only difference with respect to the MWS model remains substantially the expression of $\delta_{\alpha }$ as a function of α. As can be observed in Table S12, the equivalent aspect ratio calculated by using Equation (18) is three times higher than the one calculated by using the MWS model (Equation (11)), close to the values observed for lamellae (10–100), rather than for spherulites (0.25–4).

Other models or extensions can be found in the open literature for the description of the tortuosity in composites systems. It is, however, out of the scope of the present work to give a complete overview of them, since several bibliographic reviews exist in this concern [65,66,67]. For the current study, indeed, the presented models can be used as a solid base for the representation of the simple systems and for the analysis of possible modifications accounting for factors like orientation and shape of the impermeable phase. In the following, the results obtained using such analytical models will be compared with outcomes from simulation-based approaches, used to explore more complex geometries, which are difficult to study analytically.

## 4. Methodology

The finite volume method (FVM) used in this work is based on a tridimensional reproduction of the material with a subsequent discretization of the geometry of interest. The first step was to generate representative volume elements (RVEs), subsequently adding inclusions without overlapping. This was performed by exploring different levels of complexity, going from ordered to disordered arrays, and changing the size distribution of inclusions. MATLAB^®^ and Python algorithms were coupled to generate each RVE on Space Claim 2020 R2^®^. Geometries were then fed to Fluent 2020 R2^®^ to set the mass transport problem, minimizing the residual of the elliptical partial differential equation (PDE) associated with steady-state diffusion across a plane sheet, also known as the Laplace equation:

(19) $$0=\nabla^{2}f$$

where f is the gas fugacity and $\nabla^{2}$ denotes the Laplacian operator. In order to solve Equation (19), two Dirichlet’s boundary conditions were set at the two faces of the RVE normal to the x direction (flow direction) in order to set an arbitrary value for the gradient across the RVE.

(20) $$f\left(x=0\right)=f_{0}$$

(21) $$f\left(x=L\right)=f_{L}$$

On the other hand, Newman’s boundary of zero flux was set at each amorphous surface S, adjacent to the surface of the inclusions:

(22) $${\left[\nabla f\right]}_{S}=0$$

It is noticed that the inner surface and volume of the inclusions is excluded from the domain of interest, as they are assumed to be completely impermeable. Finally, periodic boundaries were assigned at the surfaces normal to the y and z directions to represent an infinite sheet of material:

(23) $${\left[\nabla f\right]}_{y=0}={\left[\nabla f\right]}_{y=L}$$

(24) $${\left[\nabla f\right]}_{z=0}={\left[\nabla f\right]}_{z=L}$$

Once the problem was defined, Equation (19) was solved in the whole domain to compute the effective flux in the RVE on the x direction J, using Equation (1), and the flux in the completely permeable amorphous RVE $J_{a}$ by using Equation (2). Finally, the flux reduction with respect to the pure permeable matrix was computed, in terms of $P_{r}$, trough Equation (3), by scaling J with $J_{a}$.

The same procedure, better described in Supplementary Section S.2, is repeated for different RVEs, with different levels of complexity. This method was used initially to analyze ordered arrays, to check the consistency of the results, and then extended to disordered arrays. For each geometry, a mesh analysis is also considered to ensure completely mesh independent results. The mesh size used, indeed, was the highest value able to give reliable data, considering computational domains characterized by a number of discretized volumes ranging between 10^5^ and 10^7^ depending on the microstructure considered.

Starting with ordered arrays, symmetrical RVEs were reproduced based on simple cubic (SC), body-centered (BC), and face-centered (FC) unit cells made of spherical inclusions. Spheres of the same diameter were used, reaching a maximum value of ϕ equal to 70% for FC cells, close to its maximum theoretical packing fraction (74%). Then, geometries generated by cubic inclusion placed in ordered arrays were considered, using SC and BC unit cells. Values of ϕ up to 90% were obtained for cubic inclusion, since there are no packing limitations related to this this type of microstructure. First, SC arrays made of cubes were considered, where the cubes were aligned in the flux direction. Then, BC arrays were considered, where the cubes were misaligned with respect to the flux.

**Figure 2:**
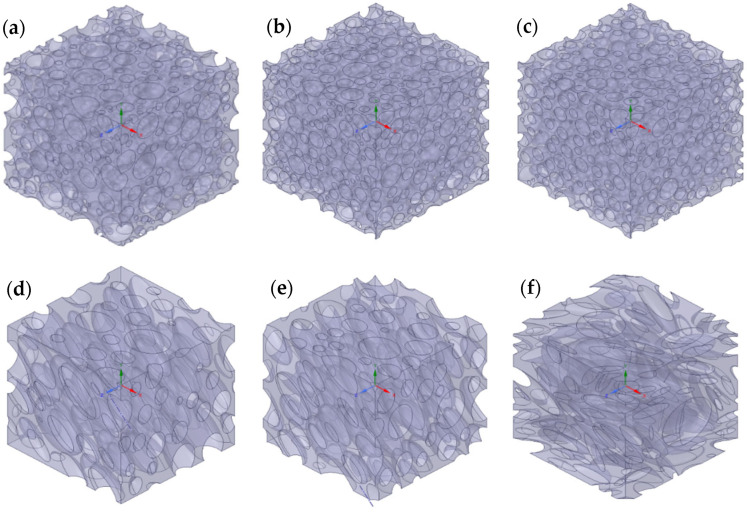
RVEs generated by different algorithms: (**a**) RVE made by random filling spheres by using N = 1000 and *ϕ* = 0.5; (**b**) RVE made by normal distributed spheres by using N = 1000 and *ϕ* = 0.5; (**c**) RVE made by lognormal distribution spheres by using N = 1000 and *ϕ* = 0.5; (**d**) RVE made by 100 oriented prolates obtained by using N = 100, *ϕ* = 0.4, α = 0.25 and θ = 60°; (**e**) RVE made by 100 oriented oblates obtained by using N = 100, *ϕ* = 0.4, α = 0.25 and θ = 60°; (**f**) RVE made by randomly oriented oblates obtained by using N = 100, *ϕ* = 0.4 and α = 4.

A finite number of spheres N was randomly arranged in a periodic cubic RVE with a fixed volume by using an RSA algorithm. Additionally, to investigate higher volume fractions, spheres of different radii were also considered. This approach allowed us to reach values of ϕ of around 60% by placing the spheres in a descending order of radius, before the computational burden became too high. The limitation arises from the maximum number of spheres and the difference between the minimum and the maximum diameter, which lead to an excessively large number of finite volumes in the final computational domain. At least 10 different RVEs were generated for each value of ϕ, in order to have statistically consistent results in terms of $P_{r}$ with a relative standard deviation always lower than 1%. In this approach, it is important to monitor the dependence of the results on the number of spheres N. An analysis made on 10 equivalent RVEs revealed that the relative permeability results are similar by using N = 100 and N = 1000, indicating that such quantity does no longer depend on N above these values. An example of an RVE obtained through the random filling algorithm by using N = 1000 and *ϕ* = 50% is reported in Figure 2a.

The use of normal and lognormal distribution was also considered for the sphere radius, by choosing the same values of mean spheres diameter. The variance was increased from normal to lognormal size distribution to reach a maximum value of *ϕ*, respectively equal to 40% and 50%. Two different approaches were evaluated for each type of distribution, starting from the same probability density function. The first strategy randomized the distribution values by generating 10 RVEs equivalent in terms of probability density function and volume fraction, while the second one rounded the number of spheres obtained from the probability density function to the nearest integer, to produce 10 equivalent RVEs with the same discretized distribution and volume fraction, but different spatial arrangement of the spheres. The second approach led to better packing efficiency and lower standard deviations of the relative permeability, thus allowing to decrease computational time and cost, even if the average relative permeability calculated was roughly the same for both cases. For this reason, the “rounding” strategy was used for the following simulated microstructures, obtained by changing aspect ratio and shape. More details on such analysis are reported in Supplementary Section S.2, together with graphical examples shown in Figures S3 and S4, for normal and lognormal distributions, respectively. Two examples of RVEs obtained from normal and lognormal distribution, by using N = 1000 and *ϕ* = 50%, are reported in Figure 2b,c, respectively. Also, in this case, a different number of spheres were considered to build the computational domain. Again, the difference in terms of relative permeability between N = 100 and N = 1000 was negligible, but only by using N = 1000, it was possible to reach a maximum volume fraction of 50%; for N = 100, indeed, the maximum volume fraction obtained was 50%. Despite this limitation, N = 100 was kept for the next simulated microstructures obtained by changing aspect ratio, orientation and shape, in order to minimize the computational cost.

In order to investigate the effect of the crystallite aspect ratio α on the effective permeability, periodic RVEs made by prolates and oblates with the symmetry axis aligned perpendicular to the flux were reproduced. Two types of geometry were adopted in this case. On one hand, the same geometries previously meshed were scaled by stretching the RVEs in the x direction for prolates and in y and z directions for oblates to obtain the desired aspect ratio. This approach indeed allowed us to maintain the same distribution of the particles, thus making possible a direct comparison between the different geometries where only the aspect ratio was modified, without changing the number of nodes. In addition to that, to check the consistency and generality of the previous approach, new cubic RVEs were generated through the RSA algorithm using prolates or oblates with normal and lognormal distributions with equivalent values of mean, variance and aspect ratio. It is noted that the cases of both prolates and oblates were considered in the analysis of the effect of the aspect ratio on the relative permeability in order to check the consistency with the MWS model outside the diluted region.

Then, in order to consider the effect of the particle orientation with respect to the flux, new RVEs with N = 100 inclusions were reproduced. In each RVE, the orientation angle θ was defined as the angle between the symmetry axis of the ellipsoids and the flux direction. Initially, θ was kept equal for all the inclusions. An example of an RVE made by oriented oblates obtained from a lognormal distribution, by using N = 100, *ϕ* = 0.4, α = 4 and θ = 60°, is reported in Figure 2d. Another example of an RVE made by oriented prolates obtained from a lognormal distribution, by using N = 100, *ϕ* = 0.4, α = 0.25 and θ = 60°, is reported in Figure 2e.

In order to reproduce a macroscopically isotropic material made by microscopically anisotropic regions, θ was finally randomized. An example of an RVE made by randomly oriented oblates, obtained from a lognormal distribution, by using N = 100, *ϕ* = 0.4 and α = 4, is reported in Figure 2f.

Concerning the effect of the shape in disordered arrays, the Voronoi algorithm was coupled to RSA in order to create grain-based microstructures by starting from lognormal-distributed spheres. This algorithm takes as input a set of m seed points in a vectorial space of n dimensions. For each i-point, the algorithm defines the volume of each i-polyhedra as the space of the points that satisfy the following relation:

(25) $$V_{i}=p\in D^{n}\left|d\left(p,p_{i}\right)<d\left(p,p_{j}\right)\right|\;j\neq i,\;i=1,\ldots ,m$$

**Figure 3:**
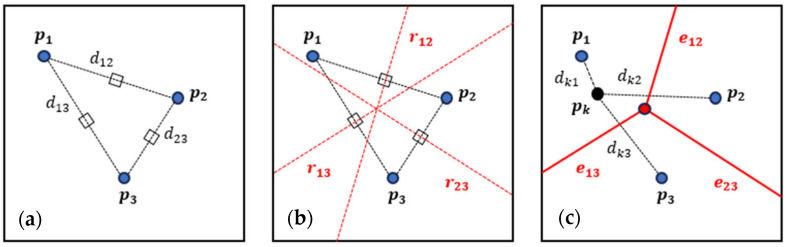
Schematization of three Voronoi algorithm steps for a simple 2D tessellation, starting from 3 seed points in a bidimensional vectorial space (n = 2). (**a**) For each couple of points $p_{i},p_{j}$ the distance $d_{ij}$ and the middle point (black squares) are evaluated. (**b**) The lines perpendicular to the distance, passing through the middle point (red dotted lines) are evaluated. (**c**) The grain areas (delimited by red edges) are defined using Equation (22). The point $p_{k}$, added for clarity’s sake, belongs to the grain generated by the seed point $p_{1}$, as the distance $d_{k1}$ between $p_{k}$ and $p_{1}$ is lower than the distances $d_{k2},\;d_{k3}$, between $p_{k}$ and all the other seed points of the domain, $p_{2}$ and $p_{3}$.

Due to the difficulty to visualize the algorithm in a 3D domain, a schematization of the algorithm in a 2D domain is depicted in Figure 3.

**Figure 4:**
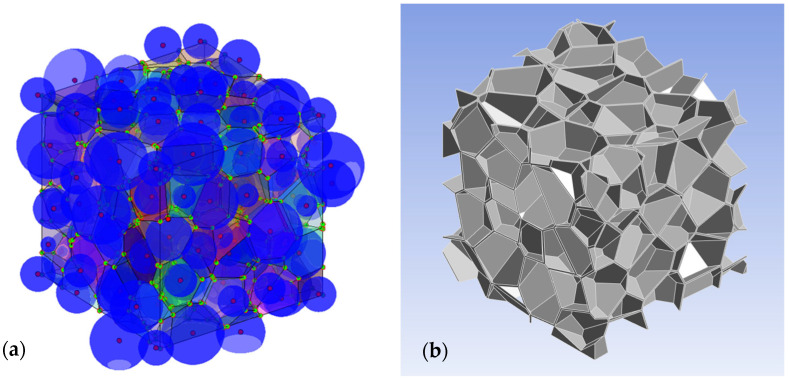
A schematization of two of the different steps involved in the process used to reproduce multiphasic materials made by different polyhedral grains embedded in a continuous matrix. In (**a**) the result of the first step is shown: the RVE made by lognormal distributed spheres (in blue) is processed, using the Voronoi algorithm, taking as input the centers of the spheres (in red), to create adjacent polyhedral grains (represented using different colors). In (**b**), the result of the second step is shown: the volume of each grain is reduced and subtracted to the whole RVE to obtain the amorphous domain (in grey).

The overall algorithm can be organized in two subsequent steps. In the first step, the centers of the spheres previously generated through RSA were fed to the Voronoi algorithm as seed points to obtain a grain-based volume, as shown in Figure 4a. Then, the volume of each polygon was reduced homogeneously, and finally subtracted to a cube, to reproduce the amorphous domain between grains in an ideal SCP, as shown in Figure 4b.

It is important to stress that lognormal distribution was not only used because it was observed in real SCPs [43], but also because the packing algorithm was more efficient than normal distribution. This approach was used to simulate a real spherulitic growth during bulk crystallization, in the assumption of a constant growth rate. The structures obtained, nonetheless, were similar to the ones reported by other authors who considered a kinetic model for the growth [91,92]. The only difference is that in this work, an ideal SCP with equally separated grains is reproduced to simplify the geometry.

Concerning the analysis of the aspect ratio, in order to increase the tortuosity of the RVEs previously obtained through Voronoi tessellation, the geometries previously generated were scaled, as performed in the case of oblates, by stretching the RVEs in x and y directions. It was not possible to obtain grains with different aspect ratios starting from centers of oblates and prolates due to the complexity of their geometry.

It is important to stress that in this case, as for all other geometries considered, 10 equivalent RVEs were generated by using the same set of parameters (N, ϕ, α, θ) in order to obtain statistically consistent results in terms of relative permeability, with a relative standard deviation always lower than 3%. The maximum relative standard deviation was calculated for each geometry, and it was found to increase from around 1%, in the case of spheres, to 3%, in the case of randomly oriented oblates and tessellations with an aspect ratio equal to 4.

## 5. Results and Discussion

**Figure 5:**
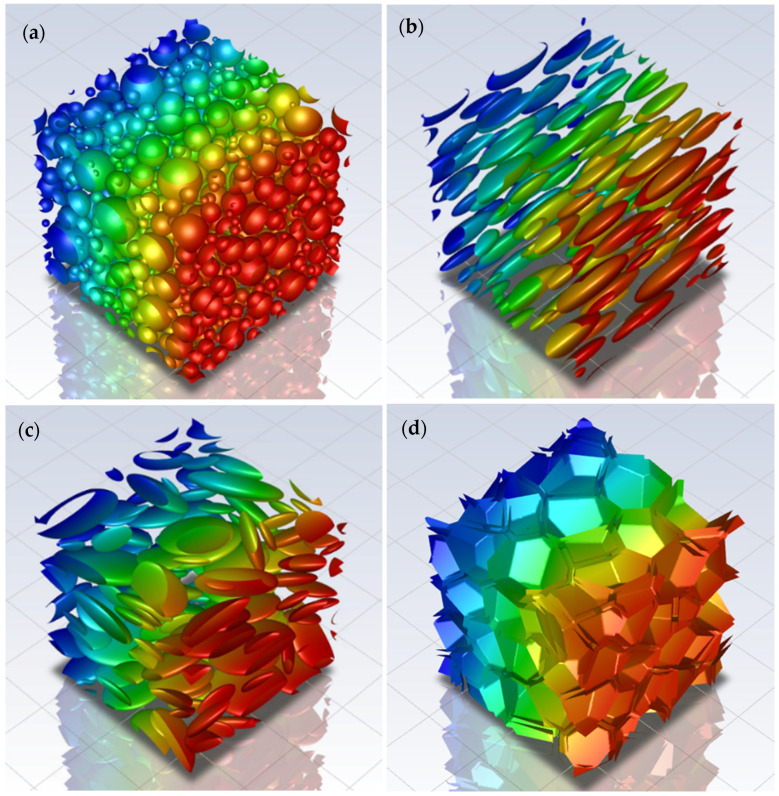
Gas fugacity profile between the upstream (red) and the downstream (blue) surfaces across different RVEs with disordered arrays, built starting with a lognormal size distribution. (**a**) RVE made by spheres, using N = 1000 and *ϕ* = 0.4. (**b**) RVE made by oriented oblates using N = 100, *ϕ* = 0.4, θ = 60° and α = 4. (**c**) RVE made by randomly oriented oblates, using N = 100, *ϕ* = 0.4 and α = 4. (**d**) RVE made by polyhedral grains obtained through tessellation algorithm using N = 100 and *ϕ* = 0.6.

The solution of the transport equation in some of the RVEs reproduced in this work is shown in Figure 5 in terms of fugacity profile (related to the gas partial pressure in the external phase) across each RVE. The average between the overall mass flux per unit area through the upstream (red) and the downstream (blue) surfaces of each RVE is computed trough FVM as $J_{eff}$ and used together with the value corresponding to the completely permeable RVE $J_{m}$ to compute the relative permeability $P_{r}$ through Equation (3). The tortuosity τ is then computed by using $P_{r}$ and the volume fraction of impermeable phase ϕ through Equation (9). The values of $P_{r}$ and τ are finally compared with the ones predicted by the different analytical models previously described. In all the following charts, the upper limit of the relative permeability according to the parallel resistance model is shown, in order to underline that all the values obtained are lower and consistent with the geometries represented. For the sake of completeness, all the numerical results obtained in this work are reported in Tables S1 in Supporting Section S.3.

### 5.1. Ordered Systems

**Figure 6:**
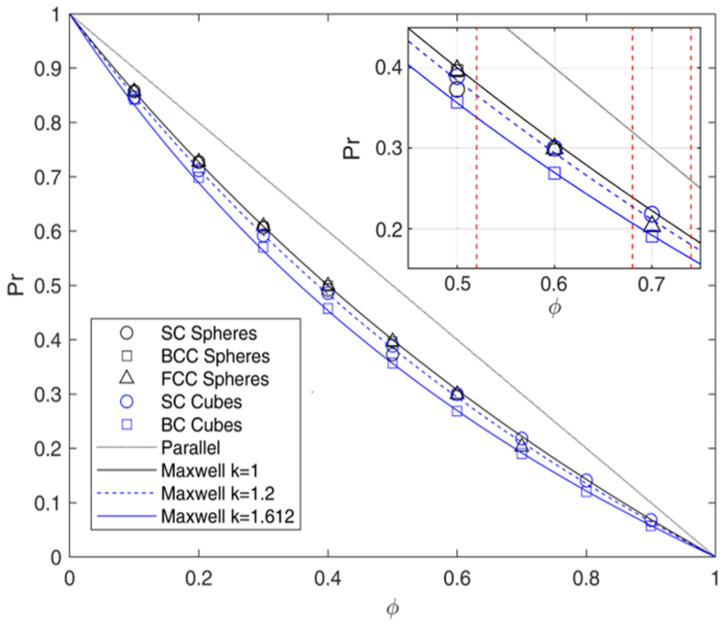
Relative permeability $P_{r}$ as a function of impermeable phase volume fraction ϕ calculated through FVM for ordered arrays of spheres (black symbols) and cubes (blue symbols). The results are compared to the Maxwell original model (Equation (10)) and the modified expression proposed in this work, to account for the deviation of the shape (Equation (26) with $\delta_{\alpha }$ = 0.5 and $k_{s}$ between 1 and 1.612). The inset shows more in detail the $P_{r}$ behavior in the limit of the different geometries maximum packing fractions (represented by dashed red lines) equal to 52%, 68%, and 74% for simple cubic (SC), body-centered (BC), and face-centered (FC) unit cells, respectively.

The analysis of the ordered system was conducted as a preliminary stage to verify the reliability of the numerical approach and its results. In Figure 6, the results of the arrays made by ordered spheres and cubes are compared to the Maxwell prediction. It can be noticed that all the results, apart from the ones obtained for BC cubes, agree with this model in the whole range of *ϕ* inspected. The same trend was reported by different authors [27,69], as shown in Figure S6, confirming the consistency of the numerical approach and the ability of this model to describe the system well above the dilute regimes, provided that the aspect ratio is close to 1. More in detail, then, the current data suggest that for the case of spheres, the arrangement does not affect the relative permeability when the volume fraction of the impermeable phase ϕ is far from the maximum packing fraction (corresponding to the case of adjacent spheres) equal to 52%, 68%, and 74% for simple cubic (SC), body-centered (BC), and face-centered (FC) unit cells, respectively. This latter result can be justified by the fact that interparticle distances are sufficiently large to ensure that the flow-line pattern around any sphere is practically undisturbed by the presence of the others, as required by Maxwell’s assumption.

On the other hand, when the geometry reaches this limit (red dashed lines in Figure 6), the reduction in the flux is enhanced with respect to the value predicted by the Maxwell model, due to the reduction in the space between spherical inclusions, so that mutual interactions between close spheres are no longer negligible. However, even in this condition, deviations with respect to Maxwell’s model are limited [27,28].

Considering the case of cubic inclusions, the situation is substantially different. First of all, the spatial arrangement of the cubes becomes relevant, as SC and BC distributions show different results. In particular, when the cubes are placed in a SC array, the relative permeability is slightly lower in the case of spheres, and with respect to the Maxwell’s model prediction, as already reported by many authors [27,28]. Interestingly, deviations from Maxwell’s model reach a maximum of around 2% at intermediate values of ϕ (30–50%), and then decrease at higher loadings.

When the cubes are placed in a BC array, the relative permeability is lower than the Maxwell’s model prediction, and relative deviations increase proportionally to the loading, reaching values of about 18% at the higher values of ϕ inspected (90%). It is clear, therefore, that the BC layout causes higher disturbance to the flow, reducing the ability of the Maxwell model to describe the flow evolution in such systems.

The linear behavior of errors suggests modifying Equation (11) to account for such effects, by including an additional constant $k_{s}$ as follows:

(26) $$\tau =1+\delta_{\alpha }k_{s}\phi$$

In Equation (26), while $\delta_{\alpha }$ is introduced to account for the effect of aspect ratio on tortuosity, $k_{s}$ can be seen as a factor that accounts for the flow alterations due to interaction between adjacent inclusion due to their shape and arrangement. Its value can be found by best fitting and is equal to 1 for spheres, about 1.2 for SC cubes and about 1.6 for cubes in the BC array, which appears to be a higher limit for this parameter when the aspect ratio is limited to 1. Interestingly, this limit is very close to the ratio between the length of a cube and the radius of a sphere with the same volume or, in other words, two times the sphericity of the cube (equal to 1.612), which once again suggests the connection of $k_{s}$ with the shape of the inclusions.

Additionally, by considering SCPs made by impermeable cubes, with $k_{s}$ = 1.612, the tortuosity obtained by using this model is always lower with respect to the ones reported in Table S12, in Supporting Section S.3 even if by using values of ϕ near 100%, considering that the fraction of grains is higher than the overall crystalline volume fraction in real SCPs. Thus, SCPs cannot be represented as ordered systems with unit aspect ratio, even considering different shapes and arrangements of the inclusions. On the other hand, Equation (26) with $k_{s}$ between 1 and 1.612 can be used to describe systems like PMCs and, for some gases, MMMs in which the filler, usually inorganic, can be considered completely impermeable with respect to the amorphous phase. The results obtained then can be easily extended to the case of system with permeable/semi-permeable dispersed phase, which will be the object of future works, considering in more detail the case of MMMs for separation applications.

### 5.2. Disordered Systems

**Figure 7:**
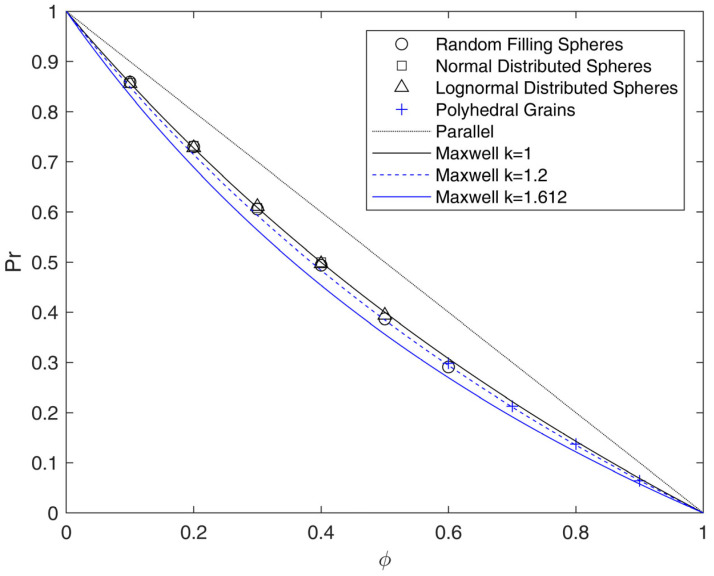
Relative permeability $P_{r}$ as a function of impermeable phase volume fraction ϕ calculated through FVM for disordered arrays of spheres (black symbols) and disordered grain-based microstructures (blue symbols). The results are compared to the Maxwell original model (Equation (13)) and the modified expression proposed in this work to account for the deviation of the shape (Equation (26) with $\delta_{\alpha }$ = 0.5 and $k_{s}$ between 1 and 1.612).

The results of disordered RVEs with randomly dispersed particles are reported in Figure 7. The cases of spheres with different dimensions following random, normal or lognormal distribution were considered, as well as polyhedral systems obtained through tessellation approaches, in order to investigate higher values of ϕ, up to 90%.

The numerical results clearly show that for spherical inclusions, the relative permeability does not depend on the size distribution, as all the different sets of geometries were in line with results obtained for ordered structures. As for the case of all-ordered arrays, all-disordered arrays showed agreement with Maxwell’s prediction at low values of ϕ and slightly underestimated, with deviations below 5%, the relative permeability calculated by the model at values of ϕ near the maximum ones reached in this work (60% for random filling algorithm, 50% for lognormal size distribution and 40% for normal size distribution).

To further increase the maximum loading up to 90% and better mimic SCPs, RVEs made by equally spaced polyhedral grains were reproduced through tessellation algorithms, starting from the centers of lognormal distributed spheres. In order to have equally spaced grains, the distances between the faces of neighboring grains were increased by reducing homogeneously the volume of each grain. This approach caused the disappearance of smaller grains at lower loadings; for this reason, the minimum volume fraction for tessellated geometries was limited to 60%. Even in these conditions, outside the diluted range, the relative permeability of tessellated geometries is similar, or slightly lower, than the Maxwell’s prediction, and remains always above the values that can be obtained through Equation (26) for BC arrays of cubes (with $\delta_{\alpha }$ = 0.5 and $k_{s}$ = 1.612), which represent the limiting case for these structures. This result could be somewhat expected, as the shapes of the generated polyhedra, for which $k_{s}$ = 1.2 (interestingly as SC-ordered cubes) are in between those of spheres and cubes. Indeed, the random distribution, as seen above for spheres, has a minor effect on the deviation from Maxwell model.

The microstructure made by polyhedral grains, therefore, does not seem to be suitable to describe real SCPs, even by using values of ϕ near 100%, as previously observed for the case of cubes. As an example, considering as upper bound $k_{s}$ = 1.612 and ϕ = 100%, the current results lead to τ = 2.612, a value substantially lower than the ones reported in Table S12.

### 5.3. Effect of the Aspect Ratio

The gas barrier properties are closely related to the aspect ratio of the impermeable inclusions α. While crystal spherulites in SPCs usually have an aspect ratio close to one, the effect of this parameter has to be considered, in view of previous results, and because industrial processes used for the production of industrial films often cause the stretching and alignment of spherulites. Similarly, in PMCs and MMMs, the aspect ratio of impermeable inclusions may depend on the production process, considering both the initial shape of the filler as well as possible effect of agglomeration.

To tackle this, we considered ordered and disordered arrays of oblate ellipsoids as well as stretched polyhedra produced from tessellated geometries. Among the ordered systems, FC arrays were chosen for ellipsoids to reach a value of ϕ equal to 60%, near the maximum (74%). For such systems, the value of α was limited to values equal to 2 and 4, and the relative results are presented in this section. The results obtained for prolates with α equal to 0.5 and 0.25 are reported in Tables S3 and S4 in Supporting Section S.3, where the effect of the size distribution on the relative permeability was also discussed. Even if those systems are not of interest for barrier applications, they were generated to check the validity of the results against the MWS model. On the other hand, BC arrays were considered for square-based parallelepipeds, in line with what was performed in the case of cubes. Also in this case, values of α equal to 2 and 4 were used, to be consistent with the ones used for oblates.

**Figure 8:**
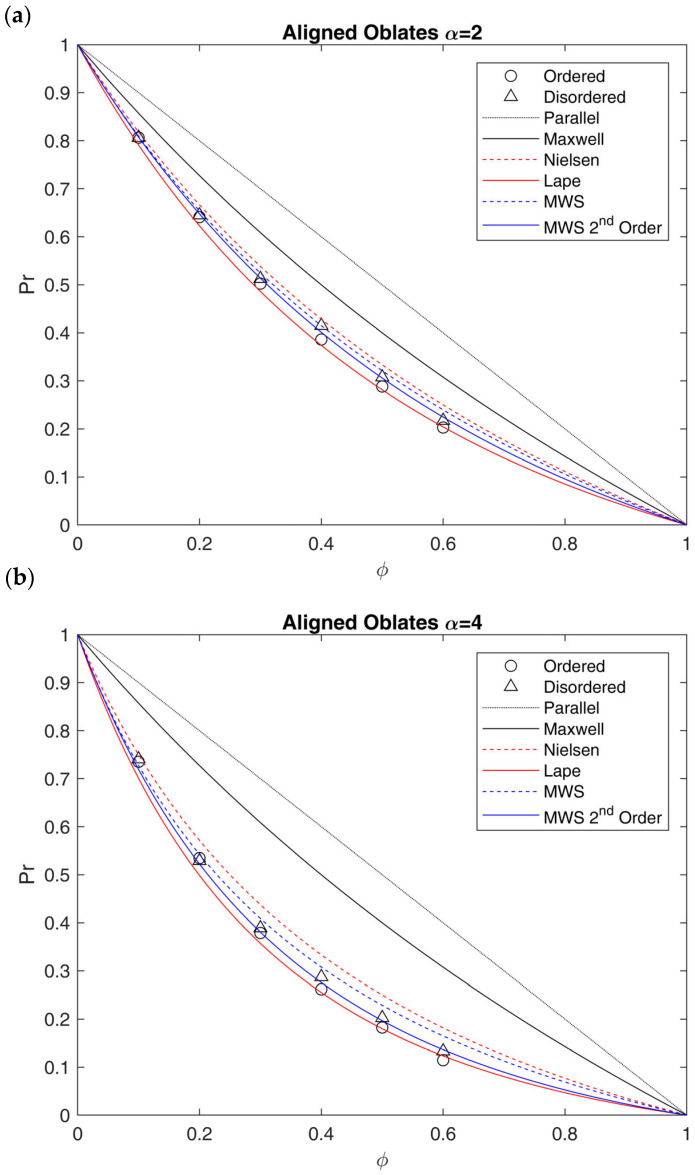
Relative permeability $P_{r}$ as a function of impermeable phase volume fraction ϕ calculated through FVM for ordered (black circles) and disordered (black triangles) arrays of oblates perpendicularly aligned with respect to the flux with different values of aspect ratio: (**a**) α=2; (**b**) α = 4. The results are compared to the ones obtained by Maxwell (Equation (10)), MWS (Equation (11)), Nielsen (Equation (12)) and Lape (Equation (15)) models, and with the modification of MWS proposed in this work, to account for the second-order term (Equation (27)).

The results for oblates placed both randomly and in ordered fashion are shown in Figure 8 and compared with different analytical models. For clarity’s sake, only the results of size distributions generated by the random filling algorithm are reported as representative of disordered arrays. All the results concerning normal and lognormal distributed oblates, which showed very limited deviation with respect to the random ones, can be found in Tables S5 and S6 in Supporting Section S.3. In addition, it should be recalled that for the models chosen for comparison, namely Nielsen, MWS and Lape, the original definition of aspect ratio presented above was used. However, for models derived from 2D systems, this choice is not univocal, as other definitions of *α* for the extension to 3D arrays were also considered in the literature [25].

In RVEs made by oblates, the effective tortuosity is definitely affected by aspect ratio and, in a more limited way, by the spatial arrangement. In this case, indeed, while the difference in the aspect ratio is more marked, ordered structures are slightly more efficient in terms of flux reduction with respect to random arrays, which ultimately are characterized by diffusional shortcuts which lead to lower tortuosity and higher relative permeability [25,26].

Comparing the results of RVEs made by oblates to the ones predicted by different analytical models, it is possible to conclude that the Nielsen model (Equation (12)) overestimates $P_{r}$ and underestimates τ in the overall range of ϕ, while MWS (Equation (11)) does it only for ϕ > 20%. This confirms the inability of the first model to describe the system, likely due to the difference in the shape between 2D rectangular lamellae and 3D ellipsoids, as well as the limitation to dilute systems for the second one.

Indeed, when the loading increases, there is a clear drift of the results towards the Lape model predictions (Equation (15)), suggesting that, even for this low aspect ratio, the linear dependence between τ and ϕ is not suitable to describe the behavior, and a higher-order dependence should be considered. In order to describe the results obtained outside the diluted region (ϕ > 20%), therefore, a modification of the original MWS model was proposed in Equation (27), which includes the second-order term to better describe the data obtained in this work:

(27) $$\tau =1+\delta_{\alpha }\phi +\frac{1}{4}{\delta_{\alpha }}^{2}\phi^{2}$$

where the coefficient 1/4 comes from the analogy with the Lape model (Equation (15)), taking $\delta_{\alpha }=2/3\;\alpha$). As clearly shown in Figure 8, Equation (27) perfectly describes the modeling results for random arrays (max error on $P_{r}$ below 4%), while it slightly overestimates the ordered arrays, which are better represented by the Lape model, at least at the higher values of ϕ inspected.

**Figure 9:**
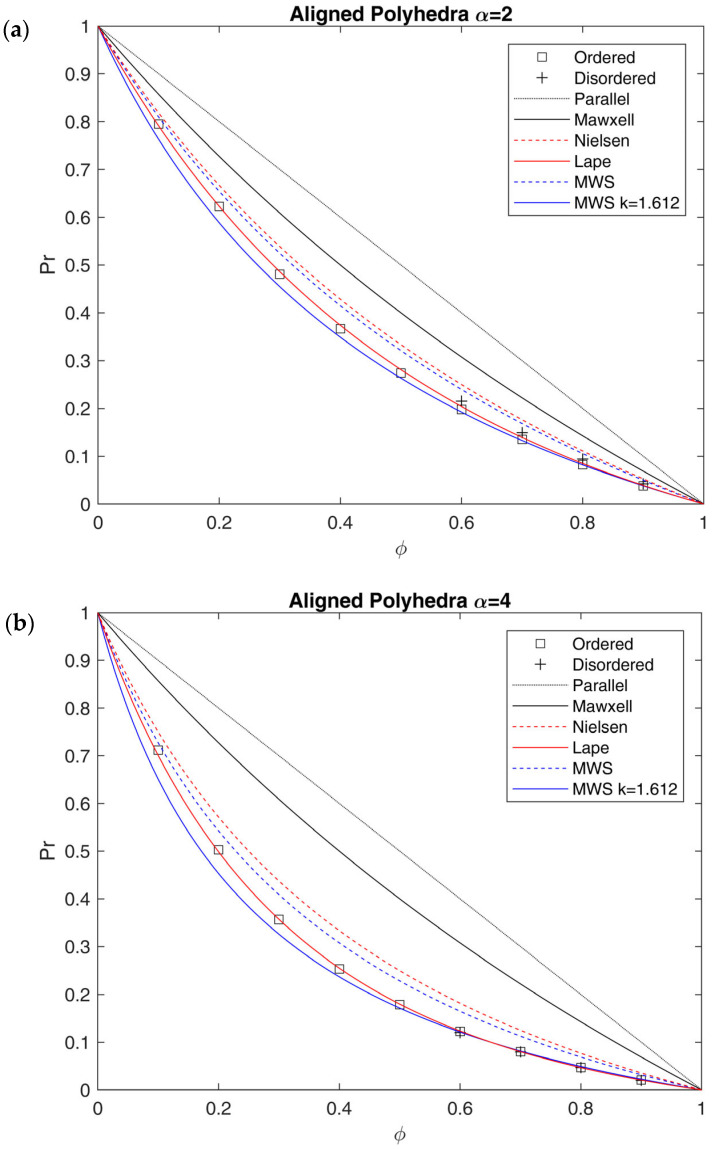
Relative permeability $P_{r}$ as a function of impermeable phase volume fraction ϕ calculated through FVM for ordered square-based parallelepipeds (black squares) and disordered polyhedral grains (black crosses) with different values of aspect ratio: (**a**) α=2; (**b**) α = 4. The results are compared to Maxwell (Equation (11)), MWS (Equation (13)), and Lape (Equation (19)) models, and with the modification of MWS proposed in this work to account for shape with $k_{s}=1.612$ (Equation (26)).

Concerning the analysis of the aspect ratio for the disordered grain-based microstructures, the results are shown in Figure 9 and compared to the case of ordered square-base parallelepipeds placed in body-centered (BC) arrays. In the same figure, we also reported the results for Maxwell (Equation (10)), MWS (Equation (11)), Nielsen (Equation (12)) and Lape (Equation (15)) analytical models. The MWS model was considered both in the original version and in the versions corrected by including $k_{s}=1.612$ (Equation (26)). On the other hand, the MWS modification for oblates, which considers the second-order term (Equation (27)), was not included, as it is not suitable for parallelepipeds, as explained below.

First of all, by comparing Figure 8 and Figure 9**,** is possible to observe that the values of $P_{r}$ obtained for ordered parallelepipeds are always slightly lower than the ones obtained for oblates, while the disordered grain-based geometries fall between the two cases, even if the differences are quite negligible. This result is in line with the trend already observed for the case of unitary aspect ratio and is substantially related to the shape of the inclusion.

In more detail, then, it is possible to observe that, in the range of aspect ratio observed, polyhedral geometries remain substantially in between the prediction of the original MWS model and the one modified with $k_{s}=1.612$ to account for the presence of sharp edges in the structure. This model indeed gives lower permeability with respect to the numerical analysis, and for values of ϕ higher than 50%, it is able to describe well the results obtained for both ordered and disordered arrays. The addition of a second-order term, therefore, seems not to be needed in this case to redefine the limit of the relative permeability obtained, even if it allows for a better fit of the simulation results.

In this regard, Equation (28), which fit oblates results, seems not effective for polyhedral inclusions, as it remains always above the Lape Model (Equation (15)), which, on the other hand, agrees very well with the results obtained in this work both for ordered square-based parallelepipeds and disordered grain-based geometries.

This latter result can be underlined by calculating the equivalent aspect ratio that is needed to describe the tortuosity calculated from experimental data, by using the Lape model (Equation (15)) and the modification of the MWS model proposed in this work to account for the shape (Equation (26)) and the 2nd order term (Equation (27)), as shown in Table S12 in Supplementary Section S.3. By considering Equation (27), the equivalent aspect ratio of the crystallites is slightly higher than the one calculated by using Equation (26) or Equation (15). In all cases, however, the values of the calculated aspect ratio are lower than the one previously calculated by using the original MWS and Nielsen models, and closer to the values measured experimentally for spherulites.

In the closure of this section, it is important to remember that the presented results assume that all the impermeable spherulites are aligned perpendicular to the flux, so that the calculated equivalent aspect ratio is always far from the real one, since the lamellae in real SCPs can be randomly or preferentially oriented. The results obtained by changing the orientation of particles are presented in the following section.

### 5.4. Effect of the Orientation

**Figure 10:**
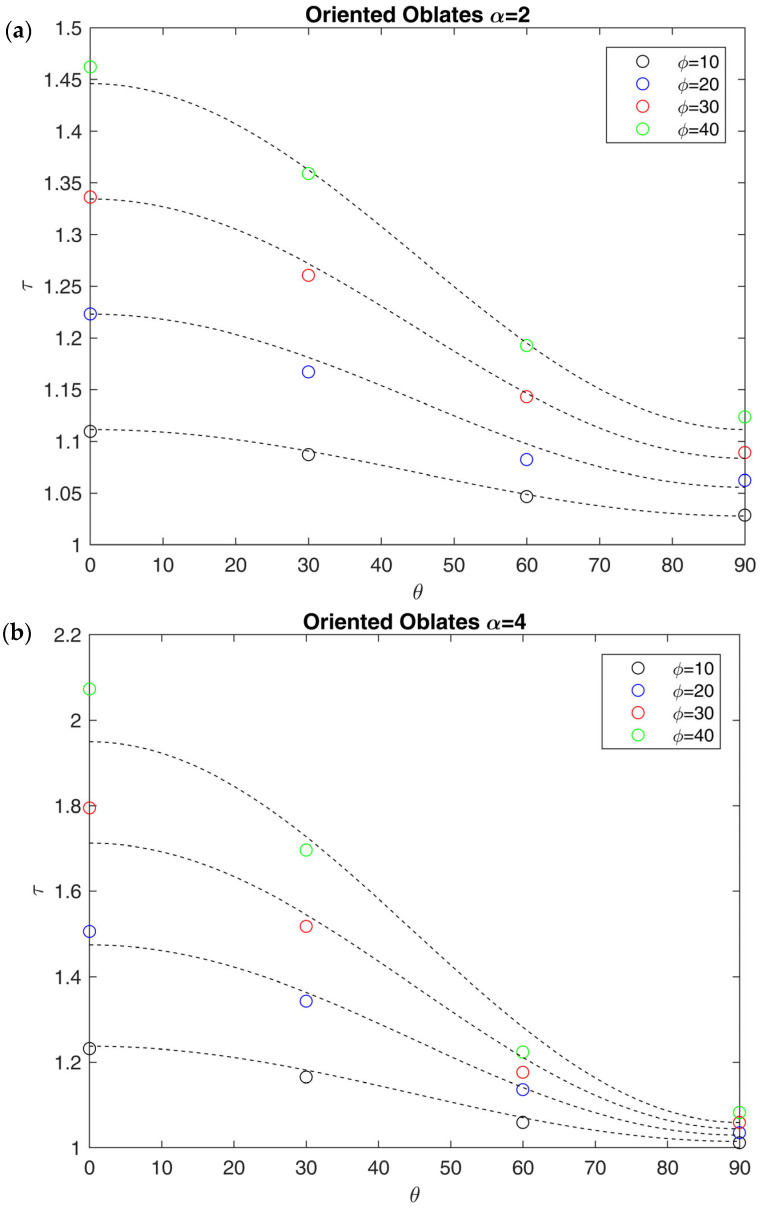
Tortuosity factor τ against orientation angle θ for lognormal distributed arrays of oblates with different values of volume fraction ϕ and aspect ratio: (**a**) α=2; (**b**) α = 4. The values calculated from FVM (colored dots) are compared to the ones obtained by using Equations (28) and (29) (dashed lines).

The influence of filler orientation was inspected in the case of ellipsoids. For parallelepipeds and tessellated geometries, orientation could not be controlled due to the difficulty of representing periodic boundaries for such arrays. The results for periodic RVEs made by spheroidal oblates oriented with different angles with respect to the flux direction are shown in Figure 10, in terms of tortuosity τ against the orientation angle θ, defined as the angle between the symmetry axis of the ellipsoids and the flux direction. Also, the effect of the orientation on oblate inclusions was investigated, and the results are shown in Tables S8 and S9 and Figure S10 in Supplementary Section S.3. For the case of prolates and oblates, the effect of the orientation of the particles was investigated varying ϕ from 10% to 40%, since the packing efficiency of the RSA algorithm decrease when θ is near 45°. The tortuosity τ was here derived from $P_{r}$ and ϕ by using Equation (10).

The main result, as clearly shown in Figure 10, is that at constant ϕ and α, τ is a sinusoidal function of θ. The same trend was observed by Bhardwaj [29], who proposed a modification of the Nielsen model to account for the orientation of the lamellae, through an orientation factor. The only difference is that the Bhardwaj model (Equations (16) and (17)) becomes equal to the Nielsen model (Equation (12)) when the lamellae are oriented perpendicular to the flux, while it is equivalent to the parallel resistance model (τ=1) when the lamellae are parallel. As a consequence, it cannot be used for the case of 3D systems like randomly distributed ellipsoids, as τ is always higher than 1 in this case. For this reason, the idea of Bhardwaj was used to modify Equation (11) in order to propose a general equation which accounts for the orientation of the impermeable phase with respect to the flux:

(28) $$\tau =1+\delta_{\alpha }f_{\theta }\phi$$

where $f_{\theta }$ is the orientation factor, which depends on the geometry and the orientation angle θ. Based on the observed bumerical results, we proposed the following expression for $f_{\theta }$, which seems indeed to be able to properly describe the behavior of oblates in the range of loading investigated:

(29) $$f_{\theta }=\frac{1}{\alpha^{2}}+\left(1-\frac{1}{\alpha^{2}}\right){cos}^{2}\theta$$

As a result, when θ=0, $f_{\theta }$ becomes equal to 1, so that Equation (29) becomes equal to the original MWS model (Equation (11)). An equivalent expression for the case of prolate ellipsoids has been also obtained and reported in Supplementary Section S.3 (Equation (S4)) for the sake of completeness. It is worthwhile to note that Equation (28) does not contain the second-order term present in Equations (15) or (27), because it is not needed to describe current results. Indeed, these second-order models, developed for inclusions perpendicularly oriented with respect to the flow, were based on two different resistances (slit resistance and tortuous path resistance), which lose significance upon rotation, making it difficult to extend them to geometries with freely oriented inclusions.

Another evident feature of these oriented systems is that the effect of orientation is greatly enhanced by the aspect ratio of the impermeable domains, as it depends on its inverse second power, according to the proposed equations. As a result, for oblates with high values of aspect ratio (α > 50), Equation (29) becomes equal to the one proposed by Bhardwaj (Equation (17)). In the limit of low aspect ratios, however, the proposed equation seems to better fit the numerical results and can be used to have a rough estimation of the effect of orientation in a multiphasic material with a spheroidal and impermeable dispersed phase. Also in this case, the analysis can be extended to the case of a permeable dispersed phase, which will be the object of future works, considering biphasic PMCs and MMMs.

**Figure 11:**
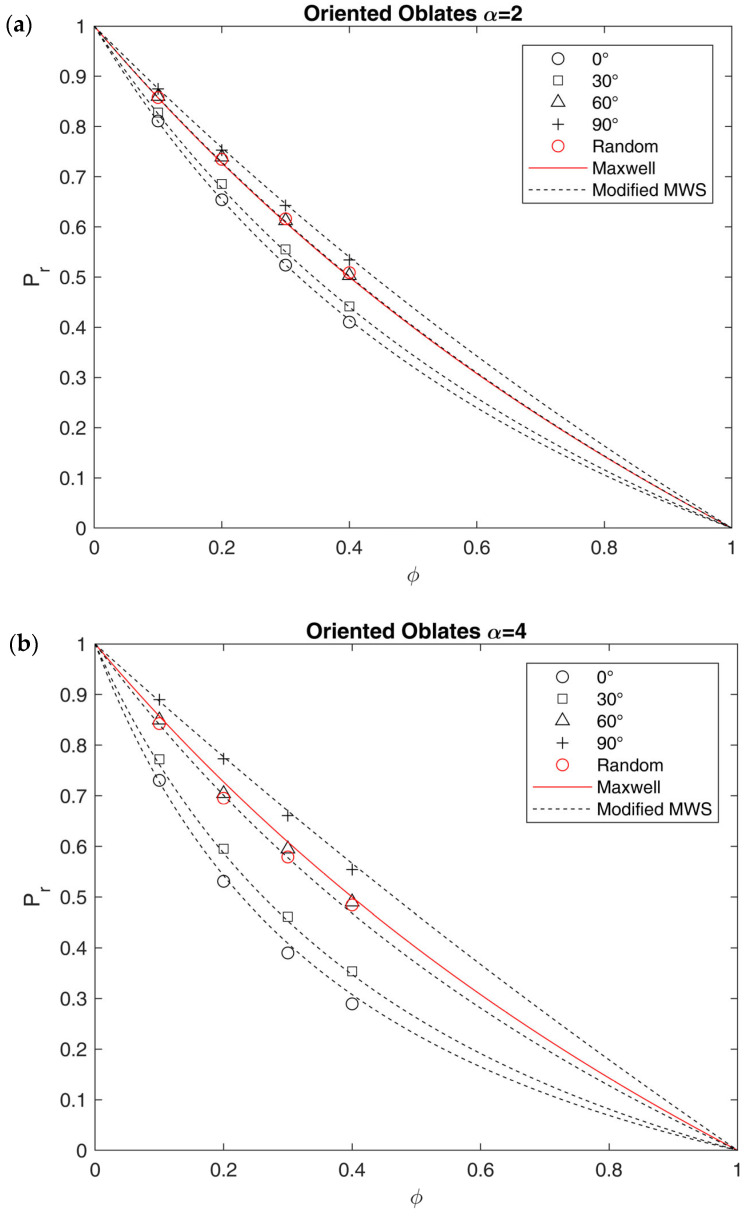
Relative permeability $P_{r}$ against volume fraction of impermeable inclusion ϕ for lognormal distributed arrays of oblates, with different values of orientation angle θ and aspect ratio: (**a**) α = 2, (**b**) α = 4. The results obtained through FVM are compared to the modified MWS model proposed in this work (Equations (28) and (29)), and with the Maxwell model (Equation (10)).

In Figure 11, the results for periodic arrays made by oblate inclusions randomly oriented with respect to the flux direction are also shown and compared to the oriented arrays, in terms of relative permeability. As expected from tortuosity data, a change in orientation greatly affects the relative permeability generally increasing it with respect to the case of inclusion oriented perpendicular to the flow direction.

An interesting result in this concern is the fact that systems with random orientation give relative permeabilities very close to those obtained with an equivalent orientation angle near to $\theta_{EQ}$ = 60°. Therefore, as said above, by randomizing the orientation of oblates, the value of the relative permeability is increased and, also for high values of α, it becomes closer to the Maxwell prediction. The latter model in particular seems quite effective in describing results obtained by oblates with α = 2, for which the value of $f_{\theta }$ at 60° is equal to 0.438, which, multiplied by $\delta_{\alpha }$ (equal to 1.12 for the MWS model with α = 2), gives a value of 0.488, very close to 0.5, the corresponding value in the Maxwell model (Equation (10)).

Again, even if such a result is still limited for the case of moderate values of α and ϕ, Equations (28) and (29) can be used to estimate the tortuosity of an ideal system, including the effect of the orientation of the impermeable particles. However, their use in obtaining an equivalent aspect ratio similar to what was made for other models in Table S12 is not trivial, as the values of $\theta_{EQ}$ should be known in advance. As a first attempt, results obtained by considering $\theta_{EQ}$ = 60°, as representative of randomly oriented systems (which better describe real SPCs) are included in Table S12. In these conditions, $f_{\theta }$ would take values between 0.438 for α = 2 and 0.258 for α = 10, causing, respectively, a two- and a four-times increase in equivalent aspect ratio for the MWS model. The results obtained with $\theta_{EQ}$ = 60° are therefore definitely higher than those observed by assuming that crystallites are perfectly aligned (Equation (11)) and again closer to the one observed in lamellae rather than spherulites. As made for the previous models, also in this case, a comparison with the literature data is present in Figure S11. In this case, considering SCPs as systems with impermeable lamellae randomly oriented with respect to the flux, a satisfactory prediction of the experimental tortuosity data retrieved in the literature [93] was made, using Equations (28,29), by using $f_{\theta }$ = 60° with the values of lamellae aspect ratio and crystalline volume fraction retrieved in the literature [41]. For clarity’s sake, a better description of the comparison can be found in Supplementary Section S.3.

## 6. Conclusions

The results obtained confirmed that the Maxwell model was able to describe most of the structure considered even outside the dilute regime (ϕ<20%) and close to the limit of maximum packing, with the only exception of the cubes in a body-centered (BC) layout. Even in this case, however, a linear relationship between ϕ and τ still exists, so that, in order to correct the deviation from Maxwell law, a shape-constant $k_{s}$ can be introduced, which takes the maximum value of 1.612 for BC arrays of cubes.

Considering the systems with moderate values of α (between 0.25 and 4), the equation proposed by Lape et al. [61] was the most effective, for the description of systems made by polyhedral inclusions, even outside diluted conditions, while a new one was proposed to extend the analytical approach proposed by Sillar et al. [56] for systems made by oblate inclusions, in order to account for the high values of ϕ, and for the orientation with respect to the flux.

In the latter case, the effect of the orientation can be attributed to the orientation factor $f_{\theta }$, which depends on the second power of α and the second power of the cosine of the orientation angle θ. In general, by decreasing the orientation angle θ, the barrier effect decreases. Similar observations can be made for systems obtained by randomizing the orientation of the inclusions. In this case, indeed, the experimental data were quite close to those characterized by θ=60°, and shifted towards the ones predicted by Maxwell.

The different models considered were used to calculate the equivalent aspect ratio needed to describe experimental permeability data in SCPs. As a result, by considering the systems as made by crystalline oblates perpendicularly aligned with respect to the flux, the equivalent aspect ratio ranges between 2 and 10, values near the ones expected for spherulites, rather than lamellae. On the other hand, by considering the systems as made by randomly oriented oblates, the actual aspect ratio of the lamellae should be used, ranging between 20 and 100, leading to values of tortuosity near the ones calculated experimentally.

## Figures and Tables

**Table 1:** Comparison between different mathematical expressions for the tortuosity factor retrieved in the literature for regular arrays of oriented impermeable inclusions in diluted conditions.

	Maxwell	Nielsen	Fricke	MWS
τ	$1+\frac{1}{2}\phi$	$1+\frac{\alpha }{2}\phi$	$1+\frac{1-m}{m}\phi$	$1+\frac{n}{1-n}\phi$
$\delta_{\alpha }$	$\frac{1}{2}$	$\frac{\alpha }{2}$	$\frac{1-m}{m}$	$\frac{n}{1-n}$

## Data Availability

The original contributions presented in this study are included in the article/Supplementary Material. Further inquiries can be directed to the corresponding author.

## Supplementary Materials

The following supporting information can be downloaded at: https://www.mdpi.com/article/10.3390/membranes15030076/s1. Figure S1: Comparison between the explicit parameters present in MWS and Fricke models as function of aspect ratio; Figure S2: Comparison between MWS, Nielsen and Fricke models in terms of aspect factor as a function of aspect ratio; Figure S3: Size distributions generated by randomizing the number of spheres with a given diameter; Figure S4: Size distributions generated by rounding the number of spheres with a given diameter to the near integer values; Figure S5: Schematization of the procedure used in order to compute the tortuosity in semicrystalline polymers as a function of microstructural parameters, through the FVM method; Figure S6: Comparison between values of relative permeability calculated with the original Maxwell model and ones obtained from different simulations-based approaches, including this work and open literature references; Figure S7: Comparison between the relative permeability obtained in this work for ordered and disordered arrays of oblates through FVM and the one predicted by different analytical models; Figure S8: Comparison between the relative permeability obtained in this work for ordered and disordered arrays of prolates through FVM and the one predicted by different analytical models; Figure S9: Comparison between the tortuosity factor calculated in this work for random arrays of oblates and the values predicted by using the models proposed by Maxwell, MWS, Nielsen and Michaels; Figure S10: Tortuosity factor against orientation angle for lognormal distributed arrays of prolates with different volume fractions and aspect ratios; Figure S11: Tortuosity predicted through Equation (28), compared with the values obtained experimentally and with the ones obtained from a random walk analysis on spherulitic microstructures; Table S1: Results in terms of tortuosity and relative permeability, as a function of impermeable phase volume fraction for ordered arrays of spheres and cubes with different dispositions; Table S2: Results in terms of tortuosity and relative permeability as a function of impermeable phase volume fraction, for disordered arrays of spheres with different size distributions and for grain-based geometries generated from lognormal distributed spheres; Table S3: Results in terms of tortuosity and relative permeability as a function of impermeable phase volume fraction, for ordered and disordered arrays of prolates with α = 0.5; Table S4: Results in terms of tortuosity and relative permeability as a function of impermeable phase volume fraction, for ordered and disordered arrays of prolates with α = 0.25; Table S5: Results in terms of tortuosity and relative permeability as a function of impermeable phase volume fraction, for ordered and disordered arrays of oblates with α = 2; Table S6: Results in terms of tortuosity and relative permeability as a function of impermeable phase volume fraction, for ordered and disordered arrays of oblates with α = 4; Table S7: Results in terms of tortuosity and relative permeability as a function of impermeable phase volume fraction, for ordered arrays of square base parallelepipeds and disordered arrays of polyhedra; Table S8: Results in terms of tortuosity and relative permeability as a function of impermeable phase volume fraction, for disordered arrays of oriented and randomly oriented prolates with α = 0.5; Table S9: Results in terms of tortuosity and relative permeability as a function of impermeable phase volume fraction, for disordered arrays of oriented and randomly oriented prolates with α = 0.25; Table S10: Results in terms of tortuosity and relative permeability as a function of impermeable phase volume fraction, for disordered arrays of oriented and randomly oriented oblates with α = 2; Table S11: Results in terms of tortuosity and relative permeability as a function of impermeable phase volume fraction, for disordered arrays of oriented and randomly oriented oblates with α = 4; Table S12: Equivalent aspect ratio calculated by using experimental data and tortuosity values from the literature by using different analytical models [24,27,30,41,54,55,56,69,72,83,84,93].

## Abbreviations

The following abbreviations are used in this manuscript:

BC	Body-Centered
FC	Face-Centered
FVM	Finite Volume Method
HDPE	High-Density Polyethylene
MMMS	Mixed Matrix Membranes
MWS	Maxwell, Wagner, Sillars
PA6	Polyamide 6
PDE	Partial Differential Equation
PEO	Polyethylene Oxide
PMC	Polymer Matrix Composite
RAF	Rigidified Amorphous Fraction
RSA	Random Sequential Adsorption
RVE	Representative Volume Element
SC	Simple Cubic
SCP	Semicrystalline Polymer
SEO	Styrene-Ethylene Oxide
