# Supplementary material for: Modelling Gas Transport in Multiphasic Materials: Application to Semicrystalline Membranes
Source: Membranes (Basel). 2025 Mar 2;15(3):76. doi: 10.3390/membranes15030076 (PMC11944189; doi:10.3390/membranes15030076)
Supplement: Supplementary file 1 [file membranes-15-00076-s001.zip › membranes-3444742-supplementary.pdf]

membranes

Supporting Information

In the following supporting information, reference to published papers is reported referring to manuscript bibliography. Equations recalled without a "S" prior to the number also refer to the main manuscript.

S.1 Analysis of Fricke and Maxwell–Wagner–Sillars (MWS) Models

In order to demonstrate the mathematical equivalence of the MWS and Fricke models, the parameters n and m, respectively defined by Sillars [7] and Fricke [5,6] are reported as function of the aspect ratio α in the equations below. In particular, Eqs. S1a and S1b refer to the MWS model, while Eqs. S2a and S2b refer to the Fricke model, for oblates $(\alpha > 1)$ and prolates $(\alpha < 1)$, respectively.

$\alpha > 1$ [S1a]

$$n = -\frac{1}{\alpha^2 - 1} + \frac{\alpha}{(\alpha^2 - 1)^{1.5}} \log[\alpha + (\alpha^2 - 1)^{0.5}]$$

$\alpha < 1$ [S1b]

$$n = \frac{1}{1 - \alpha^2} - \frac{\alpha}{(1 - \alpha^2)^{1.5}} \cos^{-1}(\alpha)$$

$\alpha > 1$ [S2a]

$$m = \frac{1}{\sin^2 \varphi} - \frac{1}{2} \frac{cos^2 \varphi}{sin^3 \varphi} \log\left(\frac{1 + \sin \varphi}{1 - \sin \varphi}\right), \qquad \varphi = \cos^{-1} 1/\alpha$$

$\alpha < 1$ [S2b]

$$m = \frac{\cos \varphi}{\sin^3 \varphi}\left(\varphi - \frac{1}{2}\sin(2\varphi)\right), \quad \varphi = \cos^{-1} \alpha$$

Despite the different mathematical form, when these equations are plotted as in **Figure S1**, it is evident that the model parameters are related by the following relationship, making the two models strictly related:

[S3]

$$n + m = 1$$

In more detail, the two models are completely equivalent when δ_α in Eq. 11 is calculated as a function of α, as shown in **Figure S2**, where results from the Nielsen model are also included for the sake of completeness.

 https://doi.org/10.3390/membranes15030076

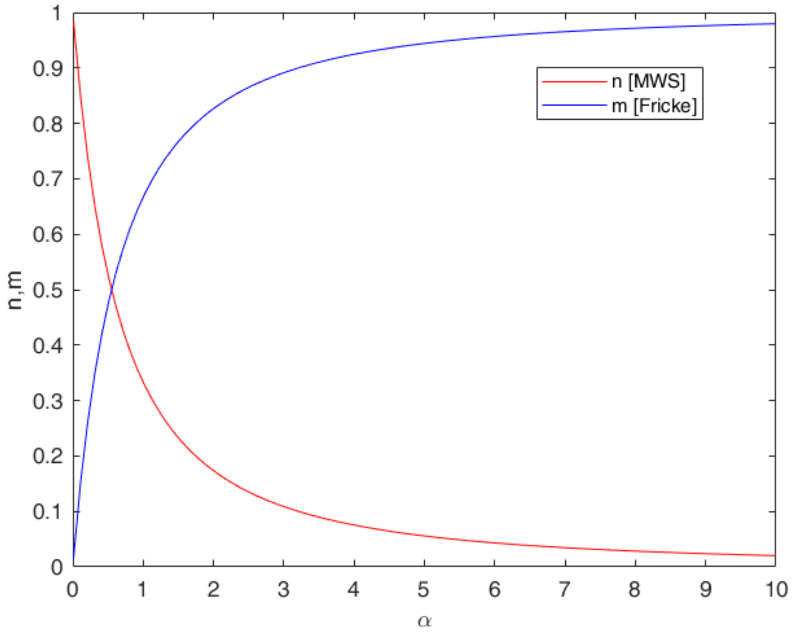

Figure S1. Comparison between the explicit parameters present in MWS and Fricke models, namely n and m, respectively, as function of aspect ratio α.

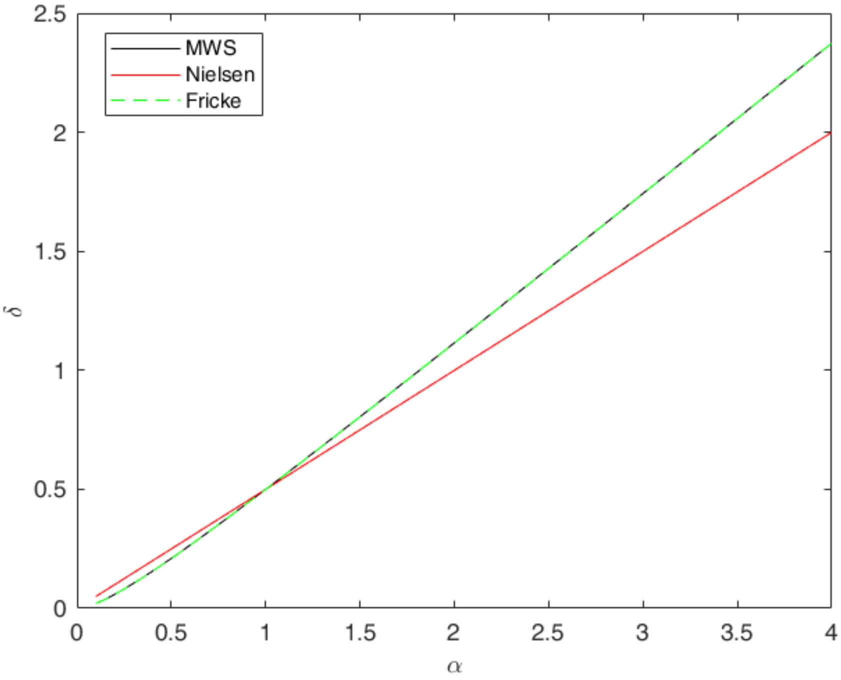

Figure S2. Comparison between MWS, Nielsen and Fricke models in terms of aspect factor δ_α as a function of aspect ratio α.

S.2 Microstructure Reproduction and Finite-Volume Method

S.2.1 Size Distribution of Sphere Radius

Considering periodic arrays made by disordered spheres, the analysis was carried out generating normal and lognormal distribution of spheres with the same mean diameter. Even if the method does not depend on the size of the computational domain, the mean diameter is here reported as the one in line with spherulites dimensions in SCPS, equal to 20 μm. The distributions were generated from the same probability density function by randomizing the number of spheres with a given diameter or by rounding the number of spheres with a given diameter to the near integer values. The variance was increased from normal (4 μm^2) to lognormal (50 μm^2) distributions to increase the maximum volume fraction of spheres. The results of the diameter distribution obtained in the two cases are shown in **Figures S3 and S4.** As discussed in the main text, both systems give similar results in terms of tortuosity or relative permeability.

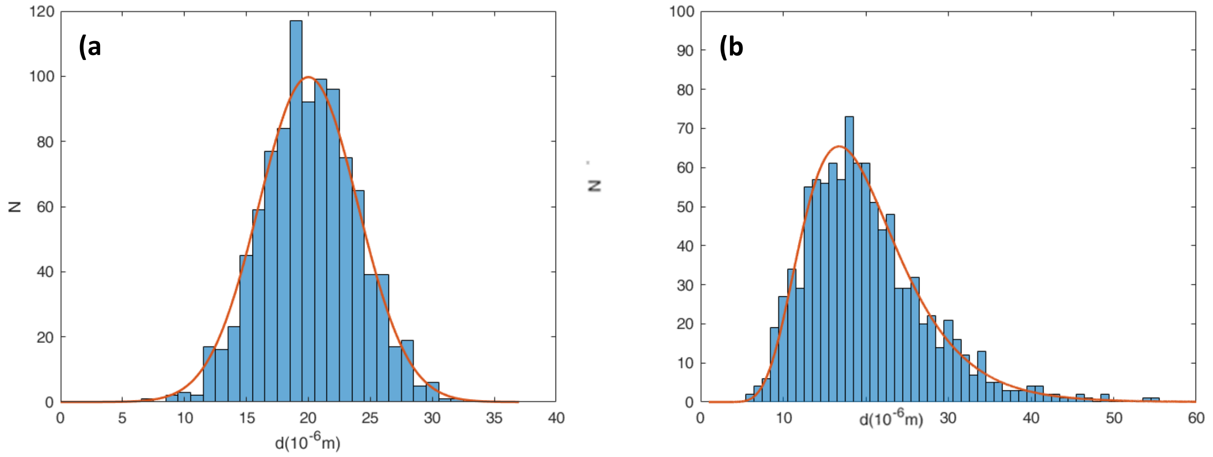

Figure S3. Size distributions generated by randomizing the number of spheres N with a given diameter d: a) normal size distribution; b) lognormal size distributions.

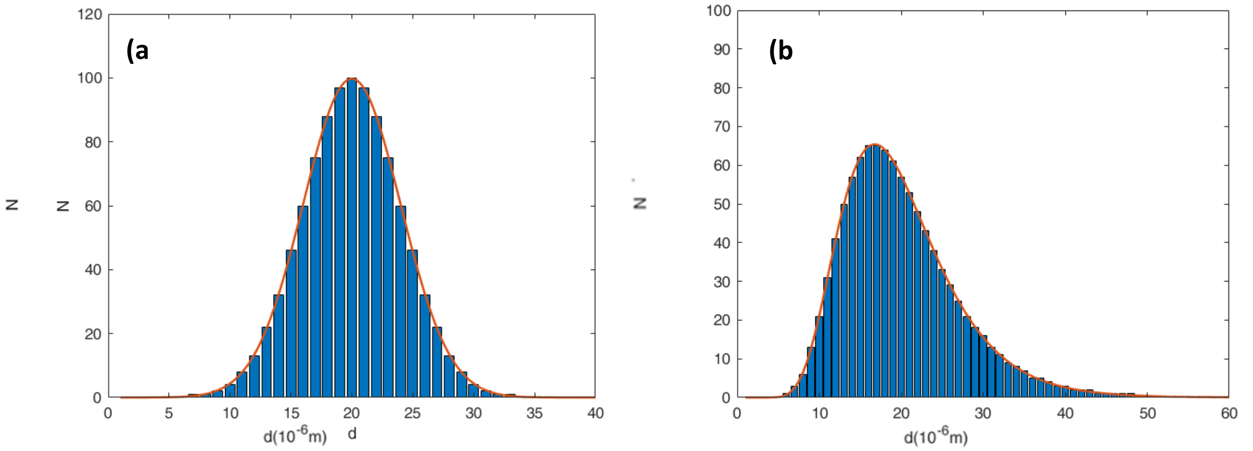

Figure S4. Size distributions generated by rounding the number of spheres N with a given diameter d to the near integer values: a) normal size distribution; b) lognormal size distributions.

S.2.2 Computational Approach based on Finite Volumes Method

The procedure is schematized in **Figure S5.** The first step was to generate representative volume elements (RVE) of the material, made by impermeable crystalline inclusions embedded in a continuous matrix, through random sequential adsorption (RSA) algorithm. The original MATLAB® algorithm proposed by Tschopp [9] was able to place a certain number of ellipsoids N in a tridimensional RVE with certain dimensions L_k in space, with $k = x, y, z$, until the system

reached the desired particle volume fraction ϕ. The algorithm was modified in order to start with a generic size distribution and fixed values of N, in order to calculate the value of L_k in order to obtain the desired value of ϕ. According to this procedure, the RVE is generated starting from microstructural parameters, such as the number of inclusions N, the dimension of the RVE L_k, the dimension of the inclusion d, their volume fraction ϕ, aspect ratio α and orientation with respect to a generic k-direction θ_k, with $k = x, y, z$. In the second step, the geometry of the continuous matrix is extracted from MATLAB® and loaded as a computational domain on Space Claim 2020 R2 ®, through Phyton® algorithms. Finally, the geometry was discretized in Fluent 2020 R2® trough the default triangular meshing algorithm, including the periodic boundaries, used to represent an infinite sheet of material. The mass transport problem was then set and solved by minimizing the residual of the elliptical partial differential equation (PDE) associated with the steady-state diffusion across an infinite plane sheet. The flux across the RVE was then computed and used for the calculation of the relative permeability.

Considering the mesh size, an analysis was performed in order to reduce the computational effort by computing the flux across the RVE by decreasing the mesh size until the result was not dependent on this parameter. This procedure was performed for each type of geometry in order to determine the highest mesh size in order to have mesh-independent results, in terms of gaseous flux across the RVE, and thus relative permeability and tortuosity. Once the optimal mesh size was chosen for each type of geometry, this parameter was fixed for 10 equivalent RVE, in terms of microstructural parameters (N, L_k, d, ϕ, α, θ_k).

Considering the numerical aspects of the partial differential equation, an arbitrary fugacity gradient was set through the Dirichlet boundary conditions (Eq. 20,21), as the results obtained in terms of relative permeability and tortuosity do not depend on the size of the RVE (N, L_k, d) but only on some of the microstructural parameter chosen (ϕ_c, α, θ_k). Indeed, the relative permeability is obtained by scaling the flux in the RVE with impermeable inclusions with the flux of an RVE without inclusions, with the same dimensions. In this case, the choice of N, L_k, d influenced only the maximum packing fraction obtainable (and thus ϕ), depending on the choice of α, θ_k. For instance, to obtain a desired value of ϕ, an higher value of particles N in a small RVE (low value of L_k) leads to a low value of particle size, but the same results in terms of relative permeability were obtained with N=100 and N=10000 (with a relative standard deviation always lower than 1%), ensuring that the results are not dependent on the size of the RVE. On the other hand, higher values of α decrease the packing efficiency of the material, as values of θ_k near 45°. For this reason, the analysis on the particles orientation was limited to a maximum value of ϕ=40%.

Considering this approach, the mesh size used for each set of equivalent RVE was the highest value able to give reliable data. From the fact that the results are not dependent on the size of the RVE, and thus on the size of the elements, but only on the ratio between this two values, the authors preferred to report the number of discretized volumes (rather their dimension for each RVE generated), which range between 105 and 107 depending on the microstructure considered.

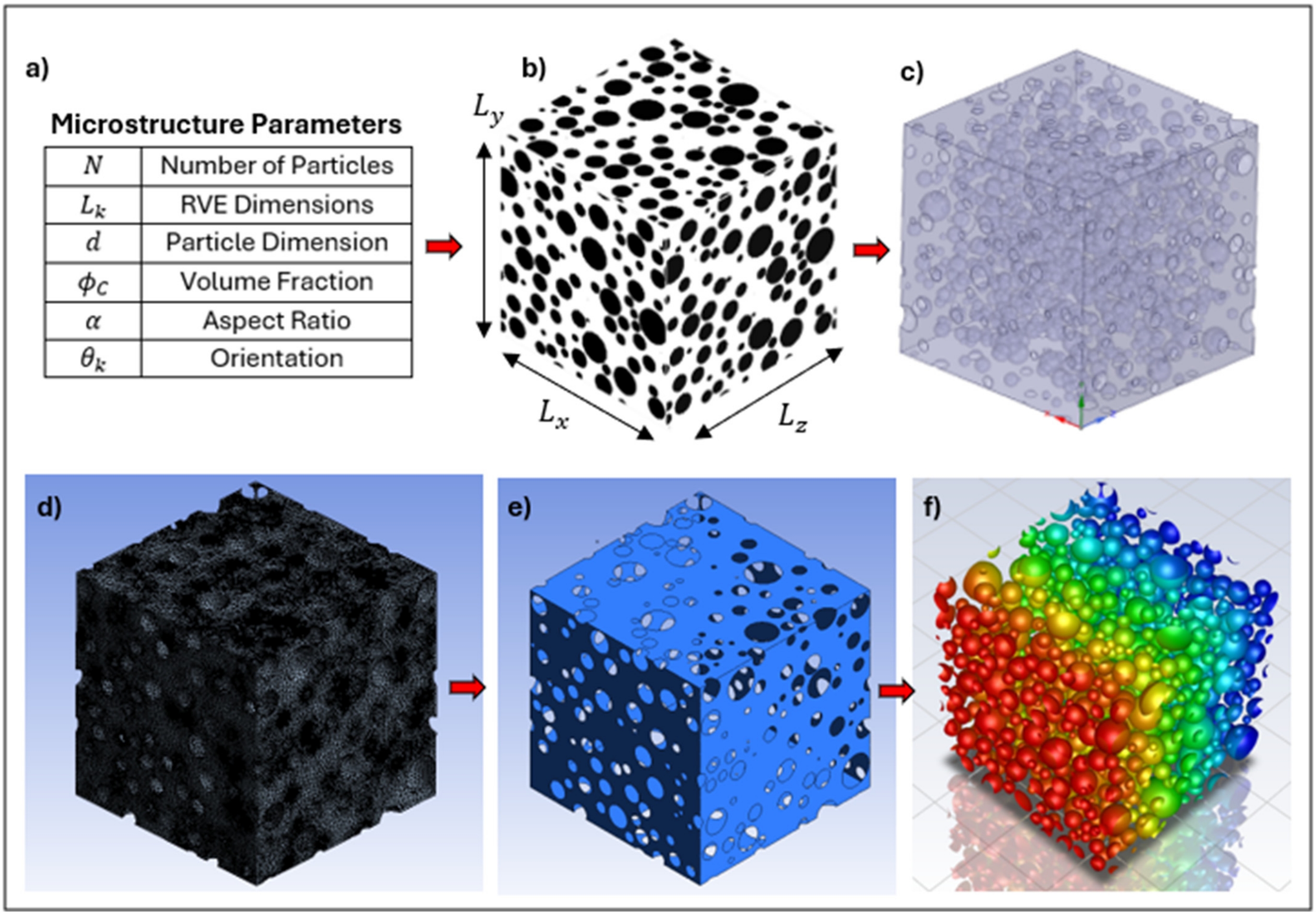

Figure S5. *Schematization of the procedure used in order to compute the tortuosity in semicrystalline polymers as a function of microstructural parameters, through the FVM method.* ***a)*** *Setting of microstructural parameters such as number of particles* N*, RVE dimensions* L_k*, particle dimension* d*, volume fraction* ϕ_C*, aspect ratio* α *and orientation with respect to a generic k-direction* θ_k*, with* $k = x, y, z$*;* ***b)*** *Generation of the RVE through the RSA algorithm;* ***c)*** *Reproduction of the RVE in Space Claim 2020 R2®;* ***d)*** *Meshing the geometry by using Fluent 2020 R2®;* ***e)*** *Setting the mass transport problem with boundary conditions (Eq. 85-90);* ***f)*** *Solving the problem in order to compute the effective flux in the semicrystalline material* J*, to be used in Eq. 3 to calculate the relative permeability* P_r*, which is finally converted in tortuosity* τ*, through Eq. 9.*

S.3 Numerical Results and Discussion

All the results obtained in this work in terms of tortuosity τ and relative permeability P_r, as a function of impermeable phase volume fraction ϕ, as well as aspect ratio α and orientation θ are reported in **Tables S1-S11.**

Table S1. Results in terms of tortuosity τ and relative permeability P_r, as a function of impermeable phase volume fraction ϕ for ordered arrays of spheres and cubes with different dispositions: SC (simple cubic), BC (body-centered), FC (face-centered) arrays. The results are compared to the Maxwell prediction (Eq. 10).

	SC Spheres		BC Spheres		FC Spheres		SC Cubes		BC Cubes		Maxwell	
ϕ	P_r	τ	P_r	τ	P_r	τ	P_r	τ	P_r	τ	P_r	τ
0.1	0.86	1.05	0.86	1.05	0.86	1.05	0.85	1.06	0.84	1.07	0.86	1.05
0.2	0.73	1.10	0.73	1.10	0.73	1.10	0.71	1.12	0.70	1.14	0.73	1.10
0.3	0.61	1.15	0.61	1.15	0.61	1.15	0.59	1.18	0.57	1.23	0.61	1.15
0.4	0.49	1.22	0.50	1.20	0.50	1.20	0.49	1.23	0.46	1.31	0.50	1.20
0.5	0.37	1.34	0.40	1.26	0.40	1.26	0.39	1.29	0.36	1.40	0.40	1.25
0.6			0.30	1.34	0.30	1.34	0.30	1.33	0.27	1.49	0.31	1.30
0.7					0.20	1.48	0.22	1.38	0.19	1.57	0.22	1.35
0.8							0.14	1.42	0.12	1.66	0.14	1.40
0.9							0.07	1.47	0.06	1.74	0.07	1.45

Table S2. Results in terms of tortuosity τ and relative permeability P_r as a function of impermeable phase volume fraction ϕ, for disordered arrays of spheres with different size distributions and for grain-based geometries generated from lognormal distributed spheres, compared to the Maxwell prediction (Eq. 10).

	Random Filling		Normal		Lognormal		Tesselation		Maxwell	
ϕ	P_r	τ	P_r	τ	P_r	τ	P_r	τ	P_r	τ
0.1	0.86	1.05	0.86	1.05	0.86	1.05			0.86	1.05
0.2	0.73	1.10	0.73	1.09	0.73	1.10			0.73	1.10
0.3	0.61	1.16	0.61	1.15	0.61	1.15			0.61	1.15
0.4	0.49	1.21	0.50	1.20	0.50	1.21			0.50	1.20
0.5	0.39	1.29			0.39	1.27			0.40	1.25
0.6	0.29	1.37					0.30	1.34	0.31	1.30
0.7							0.21	1.41	0.22	1.35
0.8							0.14	1.46	0.14	1.40
0.9							0.06	1.55	0.07	1.45

Table S3. Results in terms of tortuosity τ and relative permeability P_r as a function of impermeable phase volume fraction ϕ, for ordered and disordered arrays of prolates with α = 0.5. The results are compared to the MWS prediction (Eq. 11).

	Face Centered		Random Filling		Normal		Lognormal		MWS	
ϕ	P_r	τ	P_r	τ	P_r	τ	P_r	τ	P_r	τ
0.1	0.88	1.02	0.88	1.02	0.88	1.02	0.88	1.02	0.88	1.02
0.2	0.77	1.04	0.77	1.05	0.77	1.03	0.77	1.03	0.77	1.04
0.3	0.66	1.06	0.66	1.06	0.66	1.06	0.66	1.05	0.66	1.06

0.4	0.56	1.08	0.56	1.07	0.55	1.09	0.56	1.08	0.55	1.08
0.5	0.46	1.09	0.46	1.10			0.46	1.09	0.45	1.11
0.6	0.36	1.11	0.36	1.12					0.36	1.13

Table S4. Results in terms of tortuosity τ and relative permeability P_r as a function of impermeable phase volume fraction ϕ, for ordered and disordered arrays of prolates with α = 0.25. The results are compared to the MWS prediction (Eq. 11).

	Face Centered		Random Filling		Normal		Lognormal		MWS	
ϕ	P_r	τ	P_r	τ	P_r	τ	P_r	τ	P_r	τ
0.1	0.89	1.01	0.89	1.01	0.90	1.00	0.90	1.00	0.89	1.01
0.2	0.78	1.03	0.78	1.03	0.79	1.02	0.79	1.01	0.79	1.02
0.3	0.68	1.03	0.68	1.03	0.69	1.01	0.69	1.02	0.68	1.02
0.4	0.58	1.04	0.58	1.04	0.58	1.03	0.58	1.04	0.58	1.03
0.5	0.48	1.04	0.48	1.04			0.48	1.03	0.48	1.04
0.6	0.38	1.05	0.38	1.04					0.38	1.05

Table S5. Results in terms of tortuosity τ and relative permeability P_r as a function of impermeable phase volume fraction ϕ, for ordered and disordered arrays of oblates with α = 2. The results are compared to the MWS prediction (Eq. 11).

	Face Centered		Random Filling		Normal		Lognormal		MWS	
ϕ	P_r	τ	P_r	τ	P_r	τ	P_r	τ	P_r	τ
0.1	0.81	1.12	0.81	1.12	0.81	1.11	0.81	1.12	0.90	1.00
0.2	0.64	1.25	0.65	1.24	0.66	1.22	0.65	1.22	0.72	1.11
0.3	0.50	1.39	0.51	1.37	0.52	1.34	0.52	1.34	0.57	1.22
0.4	0.39	1.55	0.41	1.45	0.40	1.48	0.41	1.47	0.45	1.33
0.5	0.29	1.74	0.31	1.63			0.31	1.60	0.35	1.45
0.6	0.20	1.97	0.22	1.84					0.26	1.56

Table S6. Results in terms of tortuosity τ and relative permeability P_r as a function of impermeable phase volume fraction ϕ, for ordered and disordered arrays of oblates with α = 4. The results are compared to the MWS prediction (Eq. 11).

	Face Centered		Random Filling		Normal		Lognormal		MWS	
ϕ	P_r	τ	P_r	τ	P_r	τ	P_r	τ	P_r	τ
0.1	0.74	1.22	0.74	1.22	0.74	1.22	0.72	1.25	0.90	1.00
0.2	0.53	1.50	0.53	1.51	0.55	1.46	0.54	1.49	0.65	1.24
0.3	0.38	1.85	0.39	1.80	0.40	1.74	0.39	1.78	0.47	1.47
0.4	0.26	2.29	0.29	2.09	0.29	2.10	0.28	2.13	0.35	1.71
0.5	0.18	2.74	0.20	2.47			0.20	2.51	0.26	1.95
0.6	0.11	3.49	0.13	3.00					0.18	2.19

Table S7. Results in terms of tortuosity τ and relative permeability P_r as a function of impermeable phase volume fraction ϕ, for ordered arrays of square base parallelepipeds and disordered arrays of polyhedra with α = 2 and α = 4. The results are compared to the Lape prediction (Eq. 15).

	Parallelepipeds				Tesselation				Lape			
	α = 2		α = 4		α = 2		α = 4		α = 2		α = 4	
ϕ	P_r	τ	P_r	τ	P_r	τ	P_r	τ	P_r	τ	ϕ_c	P_r
0.1	0.79	1.13	0.71	1.26					0.79	1.14	0.70	1.28
0.2	0.62	1.29	0.50	1.59					0.62	1.28	0.50	1.60
0.3	0.48	1.46	0.36	1.96					0.49	1.44	0.36	1.96
0.4	0.37	1.64	0.25	2.36					0.37	1.60	0.26	2.35
0.5	0.27	1.83	0.18	2.80					0.28	1.78	0.18	2.78
0.6	0.20	2.02	0.12	3.26	0.22	1.86	0.12	3.33	0.20	1.96	0.12	3.24
0.7	0.14	2.22	0.08	3.75	0.15	2.01	0.08	3.77	0.14	2.15	0.08	3.74
0.8	0.08	2.42	0.05	4.27	0.09	2.13	0.05	4.26	0.09	2.35	0.05	4.27
0.9	0.04	2.61	0.02	4.77	0.04	2.36	0.02	5.05	0.04	2.56	0.02	4.84

Table S8. Results in terms of tortuosity τ and relative permeability P_r as a function of impermeable phase volume fraction ϕ, for disordered arrays of oriented and randomly oriented prolates with α = 0.5.

	ϕ = 0.1		ϕ= 0.2		ϕ = 0.3		ϕ = 0.4	
θ	P_r	τ	P_r	τ	P_r	τ	P_r	τ
0	0.88	1.02	0.77	1.04	0.67	1.05	0.56	1.07
30	0.88	1.03	0.76	1.06	0.64	1.09	0.54	1.12
60	0.86	1.05	0.72	1.11	0.60	1.17	0.49	1.22
90	0.84	1.07	0.70	1.14	0.59	1.19	0.47	1.28

Table S9. Results in terms of tortuosity τ and relative permeability P_r as a function of impermeable phase volume fraction ϕ, for disordered arrays of oriented and randomly oriented prolates with α = 0.25.

	ϕ = 0.1		ϕ= 0.2		ϕ = 0.3		ϕ = 0.4	
θ	P_r	τ	P_r	τ	P_r	τ	P_r	τ
0	0.90	1.01	0.79	1.01	0.69	1.02	0.58	1.04
30	0.88	1.02	0.76	1.05	0.65	1.07	0.54	1.10
60	0.84	1.07	0.71	1.13	0.59	1.20	0.47	1.28
90	0.83	1.09	0.68	1.18	0.55	1.28	0.43	1.40

Table S10. Results in terms of tortuosity τ and relative permeability P_r as a function of impermeable phase volume fraction ϕ, for disordered arrays of oriented and randomly oriented oblates with α = 2.

	ϕ = 0.1		ϕ= 0.2		ϕ = 0.3		ϕ = 0.4	
θ	P_r	τ	P_r	τ	P_r	τ	P_r	τ
0	0.81	1.11	0.65	1.22	0.52	1.34	0.41	1.46
30	0.83	1.09	0.69	1.17	0.56	1.26	0.44	1.36
60	0.86	1.05	0.74	1.08	0.61	1.14	0.50	1.19
90	0.87	1.03	0.75	1.06	0.64	1.09	0.53	1.12
Random	0.86	1.05	0.73	1.09	0.62	1.14	0.51	1.18

Table S11. Results in terms of tortuosity τ and relative permeability P_r as a function of impermeable phase volume fraction ϕ, for disordered arrays of oriented and randomly oriented oblates with α = 4.

	ϕ = 0.1		ϕ= 0.2		ϕ = 0.3		ϕ = 0.4	
θ	P_r	τ	P_r	τ	P_r	τ	P_r	τ
0	0.73	1.23	0.53	1.51	0.39	1.79	0.29	2.07
30	0.77	1.17	0.60	1.34	0.46	1.52	0.35	1.70
60	0.85	1.06	0.70	1.14	0.59	1.18	0.49	1.22
90	0.89	1.01	0.77	1.04	0.66	1.06	0.55	1.08
Random	0.84	1.07	0.70	1.15	0.58	1.21	0.48	1.24

S.3.1 Comparison with Previous Works

To verify the reliability and consistence of the computational approach presented in this work, based on the finite volumes method (FVM), the results obtained were compared with the ones obtained through different simulation-based approach and the analytical expression proposed by Maxwell (Eq. 10). As result, the equivalence between our results, the ones obtained by Qiu *et al*. [8] through a random walk analysis and the ones obtained by Minelli *et al.* [2] through FVM, is shown in **Figure S6**. It can be seen that all the different data sets are well described by the Maxwell model, suggesting the internal consistency of the proposed numerical approach, as well as the ability of Maxwell approach to describe system behavior even outside the diluted regime.

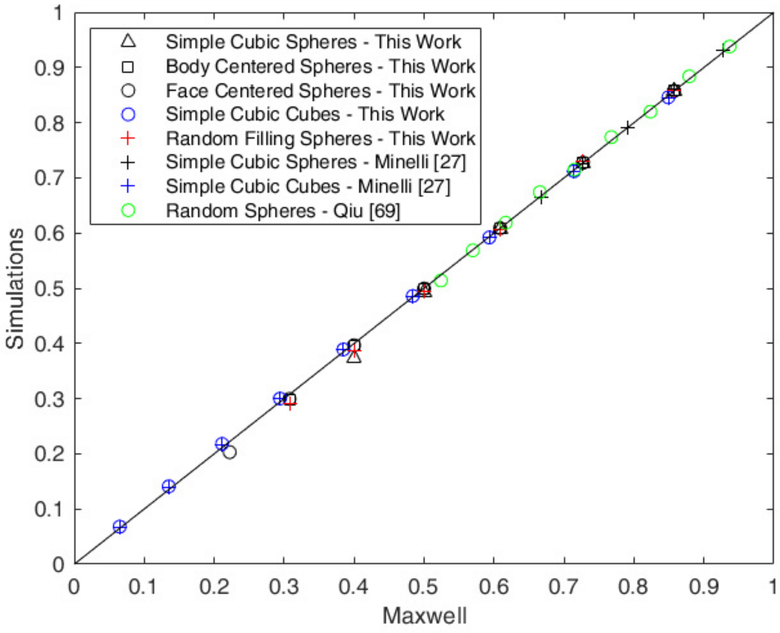

Figure S6. Comparison between values of relative permeability calculated with the original Maxwell model (Eq. 10) and ones obtained from different simulation-based approaches, including this work and the available literature [2], [8].

S.3.2 Effect of Size Distribution and Aspect Ratio for Disordered Systems

Considering the effect of the size distribution on disordered systems made by oblates and prolates, the results are shown in **Figure S7** and **Figure S8** for the different values of the aspect ratio considered.

From the figures, it is clear that the type of size distribution has an effect that is only slightly higher than the one observed for spheres, so that all disordered systems show approximately the same results. For the systems made by oblates, the relative permeability is always higher with respect to ordered systems, due to diffusional shortcuts generated by the disorder. Moreover, a deviation with respect to the MWS model is observed out from diluted conditions, for ϕ > 20%. On the other hand, the same situation is not observed for the case of prolates, for which numerical results always match MWS, with no significant difference between ordered and disordered systems. It is true that in such systems, the reduced aspect ratio pushes the results towards the parallel resistance model, which is not interesting for barrier applications. However, the latter simulations were considered as well, to validate the results obtained in this work by using the MWS model.

In addition to previous data, it has been considered useful to include in the SI some additional comparison among simulation results and other models mentioned in the text but not considered for direct comparison with the numerical results. For this reason, in **Figure S9**, the tortuosity computed in this work through FVM for random arrays of oblates is compared to the one predicted by Eq. 27 and for sake of completeness, also with the results of the prediction made by using the models proposed by Maxwell (Eq. 10), Nielsen (Eq. 12), MWS (Eq. 11) and Michaels (Eq. 18). The results show clearly that the 2nd order term (Eq. 27) better describe the system out of the diluted region, with respect to the original MWS model. On the other hand, the Nielsen model is quite unsatisfactory, even in diluted conditions, as well as the expression for the tortuosity proposed by Michaels. In particular, the latter expression gives inconsistent results as the tortuosity for oblates with α = 2 is lower than the one predicted by the Maxwell model for spheres.

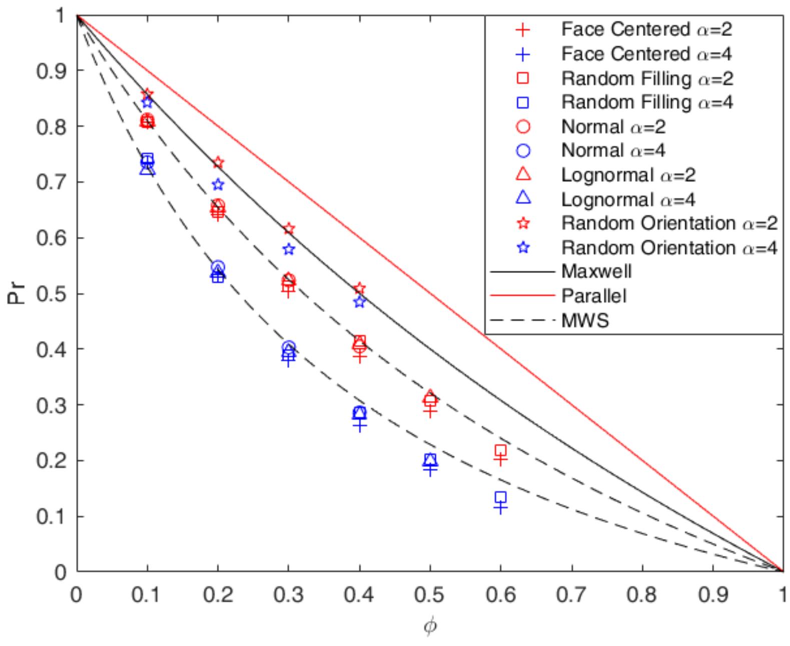

Figure S7. Comparison between the relative permeability obtained in this work for ordered and disordered arrays of oblates through FVM and the one predicted by different analytical models.

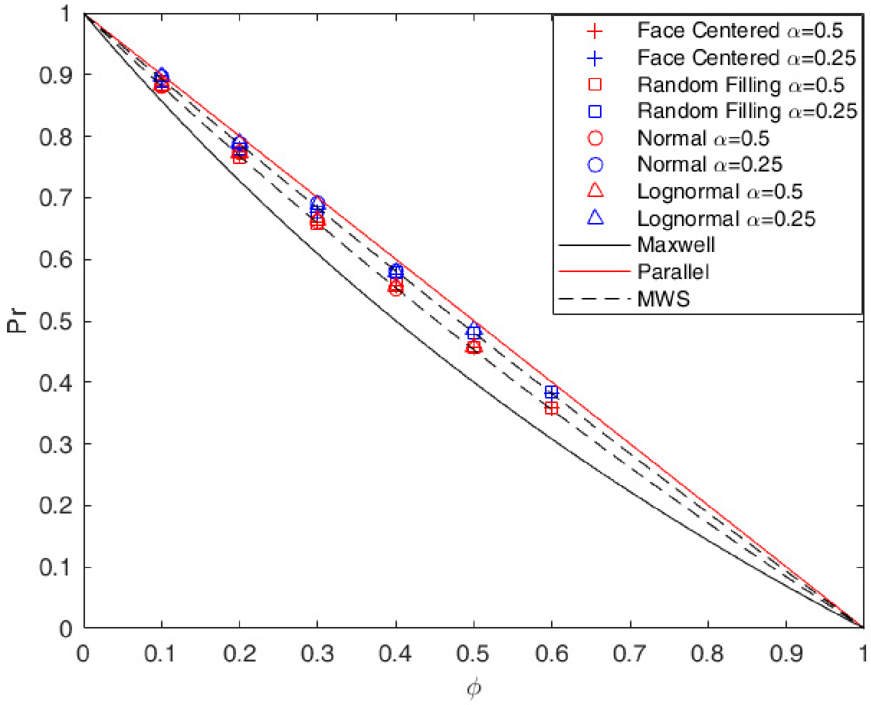

Figure S8. Comparison between the relative permeability obtained in this work for ordered and disordered arrays of prolates through FVM and the one predicted by different analytical models.

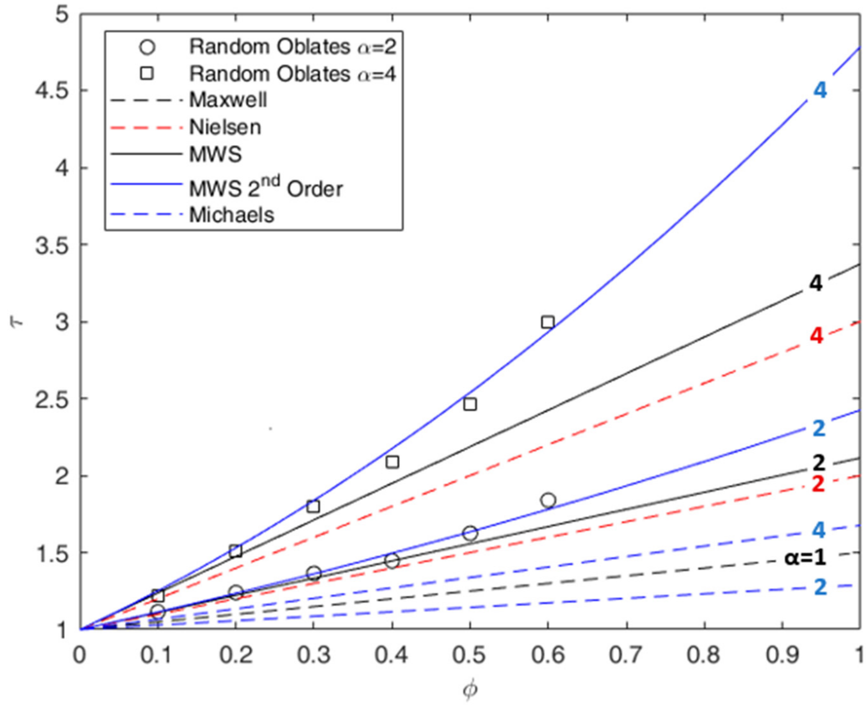

Figure S9. Comparison between the tortuosity factor calculated in this work for random arrays of oblates and the values predicted by using the models proposed by Maxwell (Eq. 10), MWS (Eq. 11) Nielsen (Eq. 12), and Michaels (Eq. 18).

S.3.3 Effect of the Orientation

The effect of the orientation of prolates was not considered in the main text as of limited interest for barrier applications. They were, however, considered and the results are shown in **Figure S10** for the sake of completeness. As in this case, an expression is proposed for the orientation factor:

$$f_\theta = 1 + \frac{2}{3}\left(\frac{1}{\alpha^2} - 1\right)(1 - \cos^2\theta) \qquad \text{[S4]}$$

As a result, when $\theta = 0$, the orientation factor becomes equal to $f_\theta = 1$, so that Eq. 28 becomes equal to the original MWS model (Eq. 11). Considering Eq. 28 together with Eqs. 29 and S4 for oblates and prolates, respectively, the model proposed can be used to represent any ideal material made by impermeable spheroidal particles embedded in a continuous matrix, considering possible variations of the aspect ratio and the orientation of the impermeable particles across the bulk. Such morphological variation can be found in SCPs and PMCs, when stress induces permanent deformation and the alignment of impermeable particles during the forming process. It is noticed that the same analysis can be extended to the case of a permeable/semi-permeable dispersed phase, which will be the object of future works, considering biphasic MMMs.

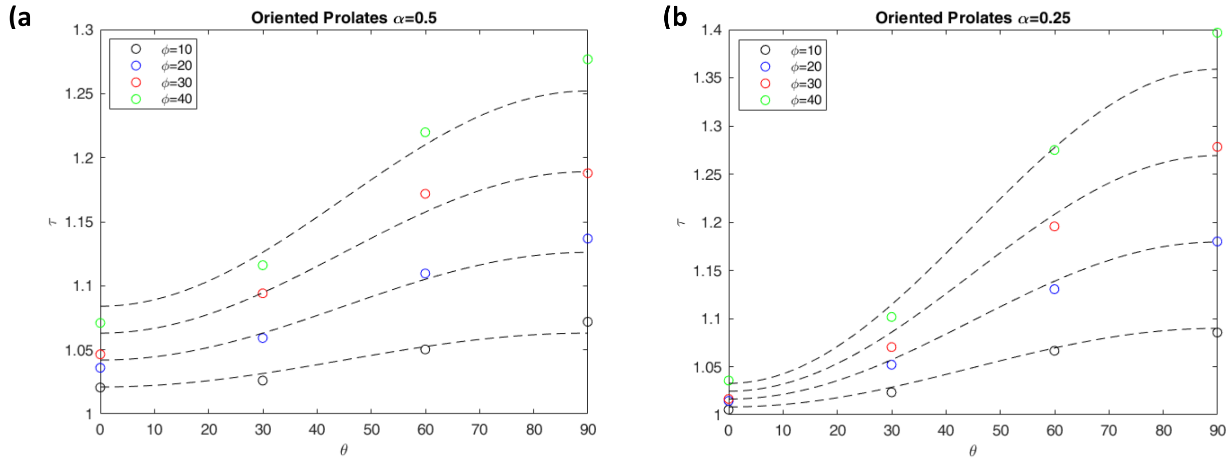

Figure S10. Tortuosity factor against orientation angle for lognormal distributed arrays of prolates with different volume fractions and aspect ratios, calculated from FVM (colored dots) and by using Eq. 28 and Eq. S2 (dashed lines)

S.3.4 Comparison with Experimental Results

The analytical models previously proposed, derived for ideal systems where spherulites are assumed to be perpendicularly oriented with respect to the flux, were used in order to estimate the equivalent aspect ratio required to describe experimental tortuosity data reported by different authors. It is noticed that the tortuosity data sets available in the literature differ not only in the experimental determination of transport coefficients, but also in the method used to estimate the tortuosity. The latter parameter cannot be directly estimated from gas permeation or sorption data, due to the lack of information on gas permeability in the amorphous polymer in the same experimental condition at which the data of semicrystalline samples were taken.

The strategy proposed in the literature by Michaels and Parker [12] was to calculate the diffusion coefficient of gases such as oxygen and nitrogen in amorphous polyethylene, as the diffusion coefficient extrapolated at ambient

temperature, by using data calculated at a temperature above the melting point. This calculation assumes that the activation energy of diffusion of gases in the amorphous phase is not dependent on the crystallinity, so that in semicrystalline systems, should be equal to the one of the melt. The tortuosity factor reported by using this method as a function of the crystalline volume fraction is shown in **Table S12**, together with the equivalent aspect ratio calculated by using different analytical models. Starting with the simplest models, the results clearly show that an aspect ratio between 4 and 12 is required for the Nielsen model (Eq. 12), while slightly lower values are required for the MWS model (Eq. 11). On the other hand, including the shape factor k_s = 1.612 in Eq. 26, the values calculated with the modified MWS model decrease with respect to the original model, towards values between 2 and 6. The results with this strategy are similar to the ones obtained by using Eq. 27, to account for the second-order term in MWS, and to the ones obtained with the Lape model (Eq. 15), considering crystallites as parallelepipeds.

On the other hand, considering randomly oriented crystallites, by using an equivalent orientation angle equal to θ_{EQ} = 60° in Eq. 16 and Eq. 29, the results are shifted towards the aspect ratio observed experimentally, and in agreement with the model proposed by Michaels and Bixler [1]. According to the results obtained, the tortuosity factor of the SCPs investigated by Hedenqvist *et al.* [4] was predicted by using Eq. 28 with θ_{EQ} = 60°, by knowing the values of α and mass crystallinity (here assumed equal to volume crystallinity ϕ) reported by the authors. Those results are then compared to the ones reported by Michaels and Parker [12] and the values predicted by the models proposed by Nillson *et al.* [3] and Pokorny *et al.* [10,11] for spherulitic microstructures in **Figure S11**, showing good agreement.

Table S12. Equivalent aspect ratio calculated by using experimental data and tortuosity values reported by Michaels and Parker [12] by using different analytical models: MWS (Eq.11), Nielsen (Eq.12), Lape (Eq. 15), Bhardwaj with θ_{EQ} = 60° (Eq. 16), Michaels (Eq.18), MWS with k_s = 1.612 (Eq.26), 2nd order MWS (Eq.27), MWS with θ_{EQ} = 60° (Eq. 28).

ϕ	τ	α(Eq.11)	α(Eq.12)	α(Eq.15)	α(Eq.16)	α(Eq.18)	α(Eq.26)	α(Eq.27)	α(Eq.28)
0.78	3.30	4.91	5.90	3.14	23.59	14.76	3.14	3.56	18.82
0.76	2.90	4.20	5.00	2.77	20.00	12.65	2.70	3.17	16.00
0.70	2.70	4.09	4.86	2.76	19.43	12.31	2.62	3.15	15.55
0.69	3.50	5.97	7.25	3.79	28.99	17.93	3.80	4.24	23.06
0.61	2.30	3.62	4.26	2.54	17.05	10.91	2.33	2.92	13.68
0.69	5.10	9.62	11.88	5.47	47.54	28.78	6.07	6.01	37.63
0.63	4.10	8.01	9.84	4.88	39.37	24.00	5.07	5.39	31.21
0.54	3.20	6.68	8.15	4.38	32.59	20.04	4.24	4.86	25.89
0.48	3.10	7.15	8.75	4.75	35.00	21.45	4.54	5.26	27.78
0.43	2.00	3.92	4.65	2.89	18.60	11.83	2.52	3.29	14.90

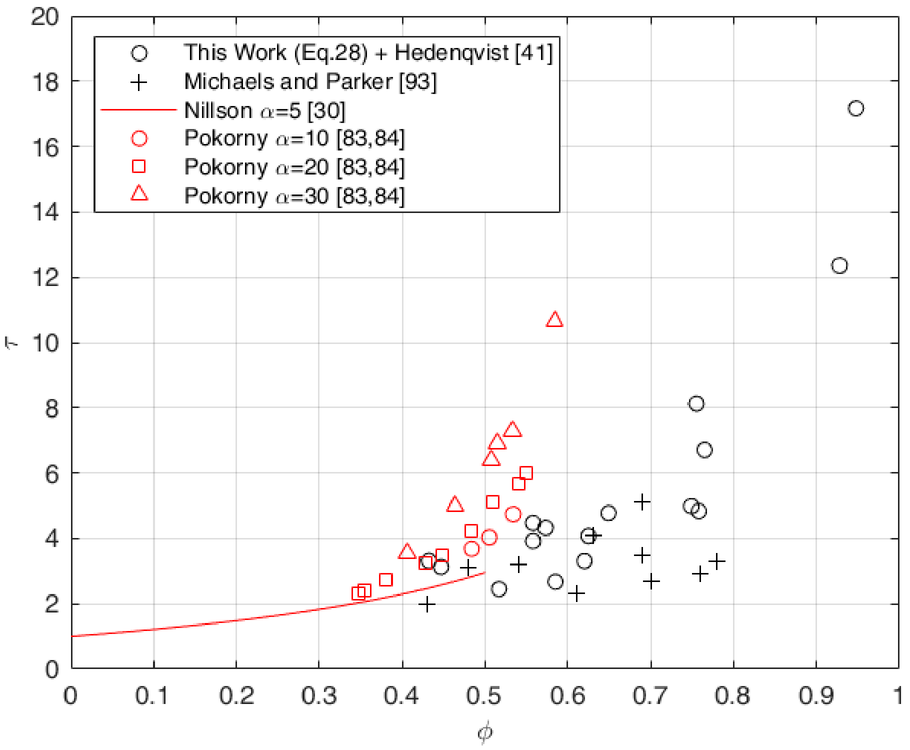

Figure S11. Tortuosity predicted by using the values of α and mass crystallinity (here assumed equal to volume crystallinity ϕ) reported by Hedenqvist *et al.* [4] through Eq. 28, compared with the values obtained experimentally by Michaels and Parker [12] and with the ones reported by Nillson *et al.* [3] and Pokorny *et al.* [10,11] obtained from a random walk analysis on spherulitic microstructures.

In conclusion, it is possible to observe that the latter approach results in a quite satisfactory prediction of the tortuosity of SCPs, as the values obtained through the proposed model, derived from an FVM analysis, are consistent with the ones determined experimentally, as well as with the ones obtained from a different simulation-based approach, based on a random walk analysis on spherulitic microstructures.

Reference

1. Michaels, A.S.; Bixler, H.J. Flow of Gases Through Polyethylene. *J. Polym. Sci.* **1961**, *50*, 413–439. https://doi.org/10.1002/pol.1961.1205015412.
2. Minelli, M.; Doghieri, F.; Papadokostaki, K.G.; Petropoulos, J.H. A fundamental study of the extent of meaningful application of Maxwell's and Wiener's equations to the permeability of binary composite materials. Part I: A numerical computation approach. *Chem. Eng. Sci.* **2013**, *104*, 630–637. https://doi.org/10.1016/j.ces.2013.09.051.
3. Nilsson, F.; Gedde, U.W.; Hedenqvist, M.S. Penetrant diffusion in polyethylene spherulites assessed by a novel off-lattice Monte-Carlo technique. *Eur. Polym. J.* **2009**, *45*, 3409–3417. https://doi.org/10.1016/j.eurpolymj.2009.09.018.
4. Hedenqvist, M.; Angelstok, A.; Edsberg, L.; Larsson, P.T.; Gedde, U.W. Diffusion of small-molecule penetrants in polyethylene: Free volume and morphology. *Polymer* **1996**, *37*, 2887–2902. https://doi.org/10.1016/0032-3861(96)89384-0.
5. Fricke, H. The electric conductivity of disperse systems, *J. Gen. Physiol.***1924**, *6*, 741–746, doi: 10.1085/jgp.6.6.741.
6. Fricke, H. The electric conductivity and capacity of disperse systems, *Physics,***1931**, *1*, 106–115, doi: 10.1063/1.1744988.
7. Sillars, R.W. The properties of a dielectric containing semiconducting particles of various shapes. *Inst. Electr. Eng.-Proc. Wirel. Sect. Inst.* **1937**, *12*, 139–155. https://doi.org/10.1049/pws.1937.0015.

8. Qiu, J.; Williams, J.; Yi, Y.B. Random walk-based stochastic modeling of diffusion in spherical and ellipsoidal composites. *Int. J. Multiscale Comput. Eng.* **2020**, *18*, 493–505. https://doi.org/10.1615/intjmultcompeng.2020033217.
9. Tschopp, M.A. *3-D Synthetic Microstructure Generation with Ellipsoid Particles*; US Army Research Laboratory: Adelphi, MD, USA, **2016**.
10. Pokorny, R.; Seda, L.; Grof, Z.; Hajova, H.; Kosek, J. Diffusion in semi-crystalline polymers. *Comput. Aided Chem. Eng.* **2009**, *26*, 961–966. https://doi.org/10.1016/S1570-7946(09)70160-9.
11. Hajová, H.; Pokorný, R.; Kosek, J. Diffusion transport in reconstructed semi-crystalline structure of polyolefins. *Macromol. Symp.* **2011**, *302*, 121–128. https://doi.org/10.1002/masy.201000073.
12. Michaels, A.S.; Parker, R.B. Sorption and flow of gases in polyethylene. *J. Polym. Sci.* **1959**, *41*, 53–71. https://doi.org/10.1002/pol.1959.1204113805.
